# Comparative Proteomic Analysis of *Hymenolepis diminuta* Cysticercoid and Adult Stages

**DOI:** 10.3389/fmicb.2017.02672

**Published:** 2018-01-15

**Authors:** Anna Sulima, Kirsi Savijoki, Justyna Bień, Anu Näreaho, Rusłan Sałamatin, David Bruce Conn, Daniel Młocicki

**Affiliations:** ^1^Department of General Biology and Parasitology, Medical University of Warsaw, Warsaw, Poland; ^2^Department of Food and Environmental Sciences, University of Helsinki, Helsinki, Finland; ^3^Witold Stefanski Institute of Parasitology, Polish Academy of Sciences, Warsaw, Poland; ^4^Department of Veterinary Biosciences, University of Helsinki, Helsinki, Finland; ^5^Department of Parasitology and Vector-Borne Diseases, National Institute of Public Health—National Institute of Hygiene, Warsaw, Poland; ^6^Department of Invertebrate Zoology, Museum of Comparative Zoology, Harvard University, Cambridge, MA, United States; ^7^One Health Center, Berry College, Mount Berry, GA, United States

**Keywords:** proteomic analysis, mass spectrometry, tapeworms, *Hymenolepis diminuta*, host–parasite interaction

## Abstract

Cestodiases are common parasitic diseases of animals and humans. As cestodes have complex lifecycles, hexacanth larvae, metacestodes (including cysticercoids), and adults produce proteins allowing them to establish invasion and to survive in the hostile environment of the host. *Hymenolepis diminuta* is the most commonly used model cestode in experimental parasitology. The aims of the present study were to perform a comparative proteomic analysis of two consecutive developmental stages of *H. diminuta* (cysticercoid and adult) and to distinguish proteins which might be characteristic for each of the stages from those shared by both stages. Somatic proteins of *H. diminuta* were isolated from 6-week-old cysticercoids and adult tapeworms. Cysticercoids were obtained from experimentally infected beetles, *Tenebrio molitor*, whereas adult worms were collected from experimentally infected rats. Proteins were separated by GeLC-MS/MS (one dimensional gel electrophoresis coupled with liquid chromatography and tandem mass spectrometry). Additionally protein samples were digested in-liquid and identified by LC-MS/MS. The identified proteins were classified according to molecular function, cellular components and biological processes. Our study showed a number of differences and similarities in the protein profiles of cysticercoids and adults; 233 cysticercoid and 182 adult proteins were identified. From these proteins, 131 were present only in the cysticercoid and 80 only in the adult stage samples. Both developmental stages shared 102 proteins; among which six represented immunomodulators and one is a potential drug target. In-liquid digestion and LC-MS/MS complemented and confirmed some of the GeLC-MS/MS identifications. Possible roles and functions of proteins identified with both proteomic approaches are discussed.

## Introduction

Similar to other parasitic flatworms, tapeworms evolved from free-living organisms; however, cestodes have probably undergone the most radical morphological changes during coevolution with their hosts (Olson et al., 2001). Cestodes became strobilated (polyzoic) organisms and abandoned their endodermal digestive tract, opting instead to absorb their food directly through their tegument. As they have complex life cycles, cestode developmental stages evolved stage-specific strategies, providing them with evolutionary success. Much more is known about cestode evolution and their successful adaptations to parasitism since Tsai et al. (2013) released a functional analysis of the genome of four tapeworm species. This was the genomic key to the cestode world and allowed an understanding of numerous aspects of their adaptive biology. We have learned that tapeworms evolved specialized detoxification pathways, metabolism that is finely tuned to rely on nutrients scavenged from their hosts and species-specific expansions of non-canonical heat shock proteins and families of known antigens. It is not known if the proteins of potential interest are expressed throughout the cestode life cycle or if they are restricted to certain developmental stages. This is the reason why proteomic analysis, including our results, is of great importance for understanding the evolutionary success of the cestode and for developing new diagnostic procedures and more effective therapies.

Tapeworms, especially the metacestode juvenile stages in the intermediate hosts, are the cause of many serious diseases of both humans and animals. Almost all adult cestodes are present only in the intestines of their definitive hosts and cause less severe symptoms than their metacestode stages (Craig and Ito, 2007). However, to adapt to digestive tract conditions, cestodes evolved a number of survival strategies.

Among tapeworms belonging to the genus *Hymenolepis*, the following three species are significant from a medical point of view: *Hymenolepis diminuta, Hymenolepis nana*, and *Hymenolepis microstoma* (Smyth, 1994). *H. diminuta* is used as a model species to investigate various aspects during cestode infection of hosts. The life cycle of this parasite includes rodents (especially rats) and humans as definitive hosts and tenebrionid beetles (*Tribolium* spp. and *Tenebrio* spp.) as intermediate hosts. The eggs pass into the external environment along with the feces of the definitive host. After eggs are ingested by the beetle, the eggs penetrate into the beetle haemocoel where the hexacanth larva, hatched from the egg, develops into a cysticercoid. This process takes about 10–14 days. When a rat or human eats the infected beetle, digestive enzymes in the stomach and duodenum cause excystation and transformation of cysticercoids, including production of proglottids with mature sexual organs. Tapeworms settle into the small intestine of the rodent and mature sexually in 2–3 weeks (Andreassen et al., 1999). Therefore, the cysticercoid stage is an important model in understanding the adaptations to invasion, while studying the adult parasite allows investigations into the mechanisms involved in interactions between the adult tapeworm and vertebrate host.

Compared with other cestode species, *H. diminuta* is rather easy to culture within laboratory hosts and may be amenable to studies based on modern molecular techniques (Rozario and Newmark, 2015). The benefits of using *H. diminuta* as a model species in laboratory tests include lack of autoinfection and low pathogenicity to the definitive host (McKay, 2010). Another advantage is its ability to infect humans; therefore, the mechanisms and adaptations observed in the rodent model should more precisely reflect the processes when the parasite infects humans.

Proteomic studies of helminths have shown new aspects in the complex coexistence of parasite and host, especially in parasitic nematodes (e.g., Yatsuda et al., 2003; Näreaho et al., 2006; Hewitson et al., 2011; Rebello et al., 2011; Bien et al., 2012) and igenetic trematodes (e.g., Jefferies et al., 2001; Bernal et al., 2004; Curwen et al., 2004; Knudsen et al., 2005; Aziz et al., 2011; De la Torre Escudero et al., 2011). With cestodes, analyses have been performed mainly on metacestode stages of Taeniidae recovered from infected animals or humans, predominantly *Echinococcus granulosus* (Chemale et al., 2003; Monteneiro et al., 2010; Aziz et al., 2011; Virginio et al., 2012), *Echinococcus multilocularis* (Wang et al., 2009; Kosik-Bogacka et al., 2014), and *Taenia solium* (Nguyen et al., 2010; Santivañez et al., 2010). Among other representatives of Cestoda, limited proteomic studies were conducted only for *Spirometra erinacei* (Kim et al., 2009) and *Mesocestoides corti* (Laschuk et al., 2011). The aforementioned studies, however, focused mainly on only one developmental stage from among the three or more stages present in the tapeworm lifecycle.

In this study, we compared the proteomes of two consecutive developmental stages (cysticercoid and adult tapeworm), which are both involved in the infection of the vertebrate host. Our findings indicated similarities and differences in the protein profiles of the metacestode and adult forms, which are suggested to affect invasion, survival, immune evasion and worm development. Future experiments should focus on the proteins of high medical potential, namely research on vaccines, new diagnostic methods, drug targets and immunomodulators as potential therapies of autoimmune disorders.

## Materials and methods

### Experimental animals

Male Lewis rats, aged about 3 months, were used as definitive hosts for adult *H. diminuta*. The rats used in the experiments were kept in plastic cages in the laboratory animal facilities of the Institute of Parasitology, PAS. They were provided feed and water *ad libitum*.

### Ethics statement

This study was approved by the 3rd Local Ethical Committee for Scientific Experiments on Animals in Warsaw, Poland (Permit number 51/2012, 30th of May 2012).

### Cultivation and collection of cysticercoid and adult stages of *H. diminuta*

Six-week-old *H. diminuta* cysticercoids were extracted from dissected *Tenebrio molitor* beetles under a microscope (magnification 100x). Isolated cysticercoids were washed five times in PBS to remove debris and were used for further protein isolation or to infect rats. Three-month-old rats (15 male rats) were infected by oral uptake of six cysticercoids of *H. diminuta* per rat. To verify the presence of adult parasites, the fecal samples of animals were examined under a microscope (magnification 400x) after 5–6 weeks from the initial infection. Rats were euthanised with 100 mg/kg intraperitoneal Tiopenthal anesthesia (Biochemie GmbH, Austria). The rat small intestines were removed immediately and adult parasites were isolated. Collected tapeworms were cultured at 37°C on a Petri dish in Eagle's media with a protease inhibitor cocktail that inhibits of serine, cysteine and metalloproteases (Roche Diagnostics GmbH, Germany) according to the procedures described by Evans (1980). *H. diminuta* adult worms and cysticercoids, handled separately, were washed up to five times with 100 mM PBS with antibiotics added to remove debris. Before protein extraction and proteomic analysis, cysticercoids and adults were stored at −80°C.

### Protein extraction

*H. diminuta* adult tapeworms and cysticercoids were suspended in lysis buffer (8 M Urea, 4% CHAPS, 40 mM Tris base) for homogenisation by sonication on ice until the suspension became clear. The homogenate was centrifuged at 54,782 × g at 4°C for 25 min to collect the supernatant containing the solubilised proteins, which were either used directly for the present study or stored at −80°C until use. The amount of total protein was measured using a Spectrometer ND-1000 UV/Vis (NanoDrop Technologies, USA). Samples used for biological replicates for in-gel and in-solution digestion were taken from independent experiments.

### One-dimensional gel electrophoresis (1-DE) and LC-MS/MS

For in-gel digestion we used three biological replicates of the adult worms (three adults worms at the same age) and two biological replicates for cysticercoids (cysticercoids in the same age ± 2 days, and minimum 2,000 cysticercoids in each sample). These protein samples were used to obtain three repetitive gels. The stained gel lanes representing each sample (replicate) were cut out in small pieces for in-gel tryptic digestion and LC-MS/MS identification. The gel protein profiles in each three replica gels were comparable. Protein concentration for both developmental stages was 10 μg/ml.

Cysticercoid and adult tapeworm somatic proteins of *H. diminuta*, separated from each other by developmental stage, were cleaned using the PlusOne SDS-PAGE Clean-Up Kit (GE Healthcare, USA). Protein samples were applied to a 12% polyacrylamide gel for 1-DE separation using 1 × TGS (0.025 M Tris, 0.192 M Glycine and 0.1% SDS) as the running buffer in denaturing conditions. To estimate the molecular weight of the proteins, a pre-stained protein standard broad-range marker (Bio-Rad, USA) was loaded on the same gel. The polyacrylamide gel was placed in a Midi-Protean Tera Cell (Bio-Rad, USA) electrophoresis apparatus. Electrophoresis was performed at 200 V constant voltage for 45 min. Separated proteins were stained with a Silver Staining Kit according to the manufacturer's protocol (Krzysztof Kucharczyk Techniki Elektroforetyczne, Poland). To digitize and analyse the repetitiveness of the protein profiles on 1-DE gels, we applied GS-800 Densitometer (Bio-Rad, USA) combined with 1-D Analysis Software Quantity 1 (Bio-Rad, USA). Gel fragments were excised manually from the repetitive gels selected lanes of the stained 1-DE. The whole silver-stained lanes were cut out, including the unstained gel regions between protein bands. Gels and the gel pieces were subjected to in-gel tryptic digestion and liquid chromatography and tandem mass spectrometry (LC-MS/MS) identification. In-gel tryptic digestion was performed by rehydration of the gel pieces with acetonitrile (ACN), reduction with 10 mM DTT in 100 mM NH_4_HCO_3_ for 30 min in 57°C and alkylation with 0.5 M iodoacetamide in 100 mM NH_4_HCO_3_ (45 min in dark at room temperature). The reduced and alkylated proteins were digested overnight with 10 ng/μl trypsin in 25 mM NH_4_HCO_3_ (Promega) at 37°C.

The recovered and purified peptide samples (20 μl in total) were subjected to LC-MS/MS identification. Samples were concentrated and desalted on a RP-C18 pre-column (Waters) and further peptide separation was achieved on a nano-Ultra Performance Liquid Chromatography (UPLC) RP-C18 column (Waters, BEH130 C18 column, 75 μm i.d., 250 mm long) of a nanoACQUITY UPLC system, using a 45-min linear ACN gradient. Column outlet was directly coupled to the Electrospray ionization (ESI) ion source of the Orbitrap Velos type mass spectrometer (Thermo), working in the regime of data-dependent MS to MS/MS switch with HCD-type peptide fragmentation. An electrospray voltage of 1.5 kV was used. Raw data files were pre-processed with Mascot Distiller software (version 2.4.2.0, MatrixScience). The obtained peptide masses and fragmentation spectra were matched to the National Center for Biotechnology Information (NCBI) non-redundant database NCBInr 20140305 (37,425,594 sequences; 13,257,553,858 residues) with a Cestoda filter using the Mascot search engine (Mascot Daemon v. 2.4.0, Mascot Server v. 2.4.1, MatrixScience). The following search parameters were applied: enzyme specificity was set to trypsin, peptide mass tolerance to ±30 ppm and fragment mass tolerance to ±0.1 Da. The protein mass was left as unrestricted and mass values as monoisotopic with one missed cleavage being allowed. Alkylation of cysteine by carbamidomethylation as fixed and oxidation of methionine was set as a variable modification.

### In-solution digestion of proteins and LC-MS/MS

For in-solution digestion we used three biological replicates for each of the developmental stages. The protein samples for in-solution digestion were extracted and pre-prepared in the same way as mentioned above. However, to remove lysis buffers from the protein sample, we used cellulose spin filters (MilliporeSigma UFC910096 Amicon® Ultra-15 Centifugal Filter Concentrator with Ultracel® 100 Regenerated Cellulose Membrane, Billerica, USA). Before use, all filters were washed with methanol and distilled water and centrifuged at 5000 × g. The protein samples were washed and diluted with 0.1 M ammonium bicarbonate and loaded onto the upper chamber of the filter. The samples were then centrifuged at 5,000 × g to pass the solution through the filter. The protein solution (500 μl) was collected in a tube and analyzed for LC-MS/MS. Protein concentration for the cysticercoid stage was 0.12 mg/ml and for the adult stage was 13.4 mg/ml. Protein solutions were subjected to standard procedure of trypsin digestion, during which proteins were reduced with 0.5 M (5 mM f.c.) TCEP for 1 h at 60°C, blocked with 200 mM MMTS (10 mM f.c.) for 10 min at room temperature and digested overnight with 10 μl of 0.1 ug/μl trypsin.

The resulting peptide mixtures were applied to a RP-18 pre-column (Waters, Milford, MA) using water containing 0.1% formic acid (FA) as a mobile phase and then transferred to a nano-HPLC RP-18 column (internal diameter 75 μM, Waters, Milford MA) using an ACN gradient (0-35% ACN in 160 min) in the presence of 0.1% FA at a flow rate of 250 nl/min. The column outlet was coupled directly to the ion source of an Orbitrap Velos mass spectrometer (Thermo Electron Corp., San Jose, CA) working in the regime of data-dependent MS to MS/MS switch. A blank run ensuring absence of cross-contamination from previous samples preceded each analysis.

### Data base searches and proteome bioinformatics

Protein identification was performed using the Mascot Search Engine (MatrixScience) with the probability-based algorithm. Multidimensional protein Identification Technology-type (MudPIT-type) and the highest number of peptide sequences (or both) were selected. The expected value threshold of 0.05 was used for analysis, which means that all peptide identifications had a <1 in 20 chance of being a random match.

Identified proteins were categorized by their molecular function, cellular component and biological processes according to gene ontology information obtained from UniProtKB (http://web.expasy.org/docs/swiss-prot_guideline.html) and QuickGO (http://www.ebi.ac.uk/QuickGO/) databases.

## Results

### DE protein separation

**Figure 1:**
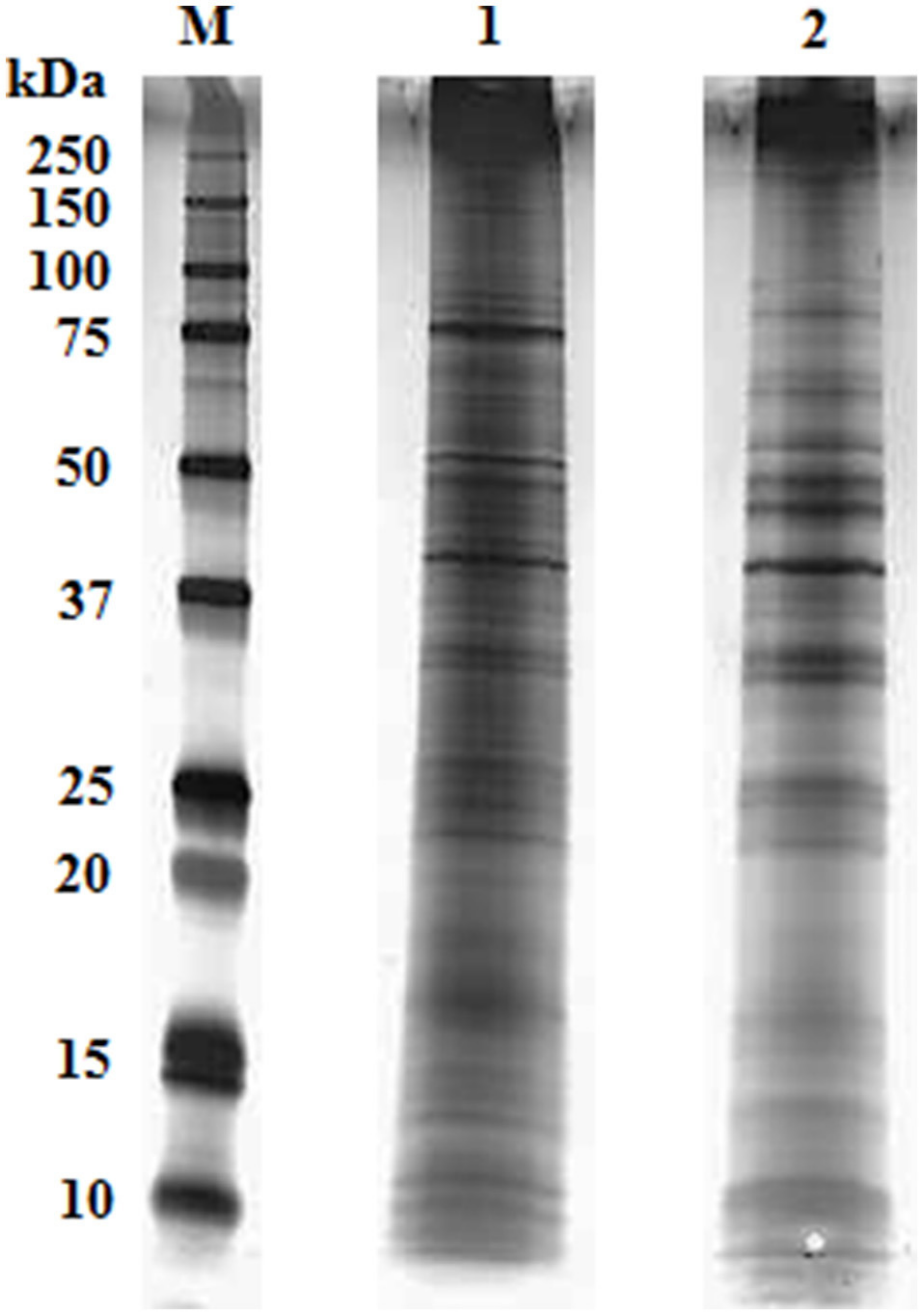
Representative overlay of silver-stained gel image of *H. diminuta* cysticercoid and adult tapeworm proteins after separation by 1-DE (M- marker, 1- cysticercoid, 2- adult tapeworm).

We obtained highly repetitive SDS-PAGE gels from biological replicates for both adult and cysticercoid stages (>99% coverage). In both samples, protein band masses ranged from <10 to over 250 kDa (Figure 1). Clear differences in the number of bands and in band optical density were observed between the adult and cysticercoid stages. The most striking differences were present in bands associated with masses of approximately 150, 90, 75, 60, 40 and 25 kDa.

### Protein identification from gels

**Figure 2:**
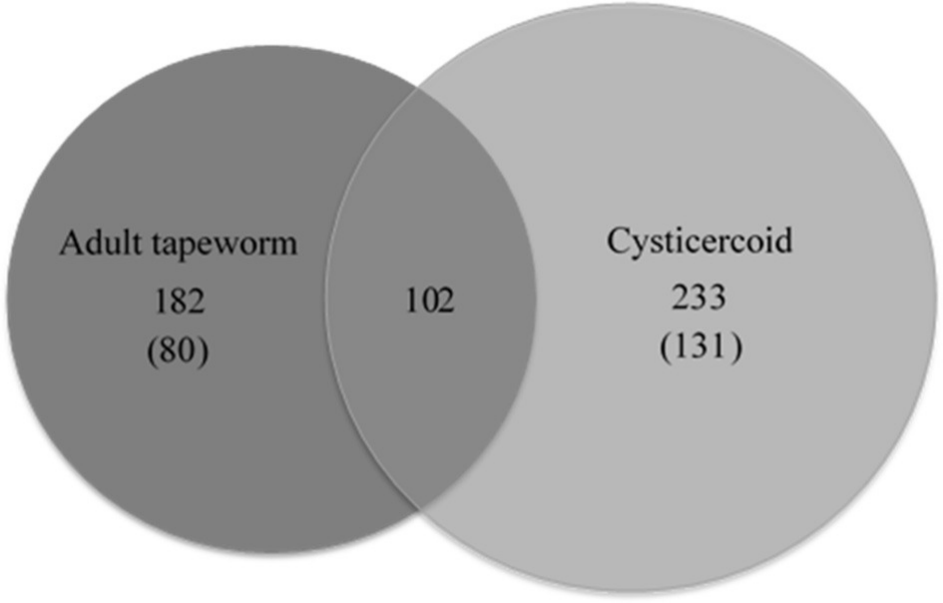
The number of proteins and common proteins identified for cysticercoid and adult tapeworm *H. diminuta*. The number of proteins identified only in either the cysticercoid or adult stage is given in brackets.

Forty-nine gel pieces were obtained from cysticercoid and 56 from adult *H. diminuta*, from each replicate. Collected gel pieces contained silver-stained protein bands and adjacent unstained regions. LC-MS/MS was used to analyse and identify proteins from the gels after in-gel tryptic digestion. Equal amounts of peptides subjected to LC-MS/MS identified 233 cysticercoid proteins and 182 adult tapeworm proteins (Figure 2, Supplementary Table 1). All proteins were identified by their homologies with proteins of other cestode species, as there is no genome or proteome of *H. diminuta* available.

Comparative analysis of proteins identified from cysticercoid and adult tapeworm indicates that these two stages share 104 proteins (Supplementary Table 1), among them proteins of medical and veterinary importance. From these proteins, 131 were observed in the cysticercoid and 80 in the adult stage. Both developmental stages shared 102 proteins, of which six represent known immunomodulators (enolase, HSP60, HSP70, paramyosin, protein disulfide isomerase, tropomyosin) and one potential drug target (enolase) (Table 1). Among proteins described as antigens, we identified 12 proteins shared by both developmental stages (Table 1). However, remarkable differences also occur in the proteomes of these two stages. Several proteins were identified from multiple bands (data not shown) that probably correspond to protein isoforms or post-translational modifications. Identified proteins were classified according to their scientific importance into the following three subgroups: antigens, immunomodulators and drug targets (Table 1). For the adult stage *H. diminuta*, we identified nine stage-specific proteins as known antigens, three immunomodulators and two drug targets (Table 1). Stage-specific antigens (four proteins), immunomodulators (three proteins) and drug targets (three proteins) were also present in the cysticercoid stage.

**Table 1:** The division of cysticercoid and adult stage proteins into three subgroups: antigens, immunomodulators and drug targets, on the basis of the available literature.

	**Name of protein**	**Adult stage**	**Cysticercoid**	**Function of protein**
ANTIGENS	14-3-3 protein	−	+	Protein binding, signaling, mitogenic signal transduction, apoptotic cell death, cell cycle control
	Actin	+	+	ATP binding
	Basement membrane-specific heparan sulfate	+	−	Malate dehydrogenase (decarboxylating) (NAD+) activity, NAD binding, transport
	Collagen	−	+	Extracellular matrix structural constituent
	Enolase	+	+	Magnesium ion binding, phosphopyruvate hydratase activity, glycolytic process
	Fatty acid binding protein	+	−	Lipid binding, transporter activity
	Filamin	+	+	Actin binding
	Fructose bisphosphate adolase	+	−	Fructose-bisphosphate aldolase activity, glycolytic process
	Glutamate dehydrogenase	+	+	Oxidoreductase activity, acting on the CH-NH2 group of donors, NAD or NADP as acceptor, cellular amino acid metabolic process
	Glyceraldehyde-3-phospate dehydrogenase	+	−	Glyceraldehyde-3-phosphate dehydrogenase (NAD+) (phosphorylating) activity, NAD binding, NADP binding, glucose metabolic process, glycolytic process
	GRP-78	−	+	Facilitating the assembly of multimeric protein complexes inside the ER
	HSP60	+	+	ATP binding, protein refolding
	HSP70	+	+	ATP binding, stress response, nucleotide-binding
	Major egg antigen	−	+	Stress response
	Myosin	+	+	ATP binding, motor activity, actin binding
	Nucleoside diphosphate kinase A	+	−	ATP binding, nucleoside diphosphate kinase activity, CTP biosynthetic process, GTP biosynthetic process, UTP biosynthetic process
	Phosphoenolopyruvate carboxykinase	+	−	GTP binding, kinase activity, phosphoenolpyruvate carboxykinase (GTP) activity, gluconeogenesis
	Phosphoglycerate kinase	+	−	ATP binding, phosphoglycerate kinase activity, glycolytic process
	Paramyosin	+	+	motor activity
	Sj-Ts4 protein	+	−	Unknown
	Spectrin alpha	+	+	Calcium ion binding
	Titin	+	−	ATP binding, protein kinase activity
	Transketolase	+	+	Catalytic activity
	Tropomyosin	+	+	Motor activity, muscle contraction, regulation of actin-myosin interaction
	Troponin	+	−	Troponin complex
	Tubulin	+	+	GTPase activity, GTP binding, structural constituent of cytoskeleton, microtubule-based process, protein polymerization
IMMUNOMODULATORS	Enolase	+	+	magnesium ion binding, phosphopyruvate hydratase activity, glycolytic process
	GRP-78	−	+	Facilitating the assembly of multimeric protein complexes inside the ER
	HSP60	+	+	ATP binding, protein refolding
	HSP70	+	+	ATP binding, stress response, nucleotide-binding
	Major egg antigen	−	+	Stress response
	Paramyosin	+	+	Motor activity
	Protein disulfide isomerase	+	+	Protein disulfide isomerase activity, cell redox homeostasis
	Pyruvate kinase	+	−	Kinase activity, magnesium ion binding, potassium ion binding, pyruvate kinase activity
	Titin	+	−	ATP binding, protein kinase activity
	Tropomyosin	+	+	Motor activity, muscle contraction, regulation of actin-myosin interaction
	Type II collagen B	−	+	Extracellular matrix structural constituent
	Zinc transportet zip8	+	−	Metal ion transmembrane transporter activity, microtubule-based process
DRUG TARGETS	14-3-3 protein	−	+	Protein binding, signaling, mitogenic signal transduction, apoptotic cell death, cell cycle control
	Aldo keto reductase family 1	+	−	Redox transformations involved in biosynthesis, intermediary metabolism and detoxification
	ATP synthase subunit alpha	−	+	Produces ATP from ADP in the presence of a proton gradient across the membrane
	ATP synthase subunit beta	−	+	Produces ATP from ADP in the presence of a proton gradient across the membrane
	Enolase	+	+	Magnesium ion binding, phosphopyruvate hydratase activity, glycolytic process
	Ndr	+	−	ATP binding, protein serine/threonine kinase activity
	Sodium/potasium- transporting ATPase	−	+	ATP binding, metal ion binding, sodium: potassium-exchanging ATPase activity

*“+,” Protein present; “−,” protein absent*.

### Functional categories of proteins by gene ontology

Proteins present in both in-gel and in-solution digestions were categorized by their molecular function, cellular component and biological processes according to gene ontology (GO) (Tables 2, 3).

**Table 2:** Functions of *H. diminuta*-identified cysticercoid and adult stage proteins according to Gene Ontology (GO) category.

**Subcategory**	**Gene ontology number (GO)**	**Number of proteins, cysticercoid**	**Number of proteins, adult stage**	**Number of common proteins, adult and cysticercoid**	**Number of proteins, cysticercoid stage characteristic**	**Number of proteins, adult stage characteristic**
**MOLECULAR FUNCTION (GO: 0003674)**						
Oxidoreductase activity	GO:0016491	22	20	13	9	7
Transferase activity	GO:0016740	23	19	11	12	8
Hydrolase activity	GO:0016787	41	34	18	23	16
Lyase activity	GO:0016829	4	4	–	–	–
Isomerase activity	GO: 0016853	4	6	3	–	3
Ligase activity	GO: 0016874	10	4	3	7	1
Signal transducer activity	GO:0004871	4	–	–	4	–
Receptor activity	GO:0004872	1	–	–	1	–
Structural molecule activity	GO:0005198	36	17	10	26	7
Transporter activity	GO:0005215	10	5	4	6	1
Lipid binding	GO:0008289	–	3	–	–	1
Phospholipid binding	GO:0005543	2	–	–	–	–
L-ascorbic acid binding	GO:0031418	1	1	1	–	–
Small molecule binding	GO:0036094	71	46	34	37	12
Ion binding	GO:0043167	101	74	52	49	22
Cofactor binding	GO:0048037	8	8	6	2	2
4 iron 4 sulfur cluster binding	GO:0051539	2	3	1	1	2
Organic cyclic compound binding	GO:0097159	87	63	42	45	21
Carbohydrate derivative binding	GO:0097367	64	42	30	34	12
Heterocyclic compound binding	GO:1901363	87	63	42	45	21
Electron carrier activity	GO:0009055	3	2	1	–	1
Antioxidant activity	GO:0016209	4	3	1	3	2
Enzyme regulator activity	GO:0030234	2	–	–	2	–
**BIOLOGICAL PROCESS (GO:0008150)**						
Cilium or flagellum-dependent cell motility	GO:0001539	–	1	–	–	1
Small GTPase mediated signal transduction	GO:0007264	–	2	–	–	1
Cell adhesion	GO:0007155	1	–	–	1	–
Signal transduction	GO:0007165	8	–	–	7	–
Nitrogen compound metabolic process	GO:0006807	51	36	21	30	15
Methyltransferase activity	GO:0008168	–	1	–	–	1
Catabolic process	GO:0009056	16	14	10	6	4
Biosynthetic process	GO:0009058	48	28	17	31	11
Cellular metabolic process	GO:0044237	78	57	33	45	24
Primary metabolic process	GO:0044238	81	59	35	46	24
Single-organism metabolic process	GO:0044710	47	40	26	21	14
Organic substance metabolic process	GO:0071704	84	62	37	47	25
Cellular process	GO:0009987	127	86	53	74	33
Single-organism process	GO:0044699	76	59	26	39	22
Response to stimulus	GO:0050896	17	7	3	14	4
Localisation	GO:0051179	16	8	4	12	4
Biological regulation	GO:0065007	20	7	4	16	3
Cellular component organization or biogenesis	GO:0071840	18	11	5	13	6
Cellular oxidant detoxification	GO:0098869	4	3	–	3	2
**CELLULAR COMPONENT (GO:0005575)**						
Proteinaceous extracellular matrix	GO:0005578	3	1	1	2	–
Gap junction	GO:0005921	–	1	–	–	1
Endoplasmic reticulum lumen	GO:0005788	1	–	–	1	–
Membrane	GO:0016020	29	20	13	16	7
Cell junction	GO:0030054	1	–	–	1	–
Macromolecular complex	GO:0032991	59	30	20	39	10
Organelle	GO:0043226	73	50	30	43	20
Organelle part	GO:0044422	40	30	18	22	12
Virion part	GO:0044423	1	1	–	1	1
Membrane part	GO:0044425	25	18	12	13	6
Cell part	GO:0044464	118	74	46	72	28
Polymeric cytoskeletal fiber	GO:0099513	8	8	4	1	4

**Table 3:** Functions of *H. diminuta* cysticercoid and adult stage proteins according to theirGene Ontology (GO) category for samples identified using in-solution digestion.

**Subcategory**	**Gen ontology number (GO)**	**Number of proteins– cysticercoid**	**Number of proteins—adult stage**	**Number of common proteins—cysticercoid and adult stage**
**MOLECULAR FUNCTION (GO: 0003674)**				
Protein disulfide isomerase activity	GO:0003756	2	–	–
Phosphopyruvate hydratase activity	GO:0004634	1	–	–
Oxidoreductase activity	GO:0016491	5	10	
Transferase activity	GO:0016740	2	7	1
Hydrolase activity	GO:0016787	12	–	–
Isomerase activity	GO:0016853	–	4	–
Ligase activity	GO:0016874	–	2	–
Nucleoside-triohosphatase activity	GO:0017111	–	7	–
Superoxide dismutase activity	GO:0004784	2	–	–
Signal transducer activity	GO:0004871	4	1	1
Receptor activity	GO:0004872	1	–	–
Structural molecule activity	GO:0005198	10	4	–
Transporter activity	GO:0005215	3	2	–
Protein binding	GO:0005515	8	7	4
Fatty-acyl-CoA binding	GO:0000062	–	1	–
Phospholipid binding	GO:0005543	1	–	–
L-ascorbic acid binding	GO:0031418	1	–	–
Lipid binding	GO:0008289	–	1	–
Small molecule binding	GO:0036094	21	25	9
Ion binding	GO:0043167	27	32	11
Cofactor binding	GO:0048037	–	7	–
Iron-sulfur cluster binding	GO:0051536	–	1	–
Organic cyclic compound binding	GO:0097159	23	29	10
Carbohydrate derivative binding	GO:0097367	20	19	9
Heterocyclic compound binding	GO:1901363	23	29	10
**BIOLOGICAL PROCESS (GO:0008150)**				
Transport	GO:0006810	–	1	–
Response to stress	GO:0006950	–	1	–
Nitrogen compound metabolic process	GO:0006807	5	9	2
Catabolic process	GO:0009056	1	6	1
Biosynthetic process	GO:0009058	4	5	–
Cellular metabolic process	GO:0044237	6	15	2
Primary metabolic process	GO:0044238	6	15	2
Single-organism metabolic process	GO:0044710	5	14	2
Organic substance metabolic process	GO:0071704	7	16	2
Cellular process	GO:0009987	18	28	9
Cellular component organization	GO:0016043	2	1	1
Single-organism process	GO:0044699	8	16	4
Localization	GO:0051179	3	–	–
Biological regulation	GO:0065007	4	5	3
**CELLULAR COMPONENT (GO:0005575)**				
Extracellular region	GO:0005578	1	1	1
Endoplasmic reticulum lumen	GO:0005788	1	–	–
Cell junction	GO:0030054	1	1	1
Organelle membrane	GO:0031090	–	1	–
Macromolecular complex	GO:0032991	11	15	4
Organelle	GO:0043226	8	12	5
Organelle part	GO:0044422	15	17	–
Membrane part	GO:0044425	5	4	1
Cell part	GO:0044464	26	31	12
Supramolecular fiber	GO:0099512	7	4	3

We established the molecular function of 186 cysticercoid and 130 adult tapeworm proteins, the cellular components for 134 cysticercoid and 85 adult proteins and proteins associated with biological processes for 151 cysticercoid and 106 adult proteins. More detailed information is shown in Table 2. In both developmental stages, the predominant molecular functions are related to binding (e.g., ion binding) and catalytic activity (hydrolase and oxidoreductase activity). Regarding biological processes, the predominant number of proteins are involved in metabolic and cellular processes. Regarding cellular components, our GO results show that the identified proteins are primarily associated with cell parts, organelles, and macromolecular complexes. The same aforementioned molecular functions were predominant among stage-specific proteins. Selected molecular functions and biological processes were identified as possibly characteristic for each developmental stage (Table 2).

### Proteins identification in-liquid digestion and their functions

LC-MS/MS was used to analyse and identify proteins after in-liquid digestion to reveal the most abundant proteins in biological sample repetitions. These included proteins with a minimum of two matching peptides in all three replicates or a minimum of three matching peptides and present in two in-solution replicates. In total, 44 cysticercoid proteins and 70 adult proteins were identified. These two stages have 21 common proteins (Supplementary Table 2). Among these proteins, 49 were identified only in the adult stage and 23 only in the cysticercoid.

After in-liquid digestion, we established the molecular function for 40 cysticercoid and 56 adult tapeworm proteins, cellular components for 28 cysticercoid and 32 adult proteins and biological processes for 21 cysticercoid and 33 adult proteins. More detailed information is shown in Table 3. The same groups of molecular functions and biological processes as identified from SDS-PAGE separation were dominant among cysticercoid and adult stage proteins.

## Discussion

### Present results with regards to *H. diminuta* adaptation to parasitism

Until now, the only analysis that characterized the proteome of the metacestode and adult developmental stages was performed by Cui et al. (2013) for *E. granulosus*. This analysis provided useful information regarding the molecular mechanisms behind host-*Echinococcus* interactions and *Echinococcus* biology. Comparative analyses of non-taeniid cestodes have been strikingly neglected, even though non-taeniid tapeworms (predominantly *H. diminuta*) are widely used in experimental cestodology (Arai, 1980; Evans, 1980; McKay, 2006, 2010; Johnston et al., 2009, 2010; Melon et al., 2010; Shi et al., 2011; Bártíková et al., 2012; Graepel et al., 2013; Hernandez et al., 2013; Cadková et al., 2014; Harnett, 2014; Kosik-Bogacka et al., 2014; Shostak, 2014; Mansur et al., 2015; Reyes et al., 2015; Woolsey et al., 2015; Zawistowska-Deniziak et al., 2017). In the present study, we identified both stage-specific and common proteins of *H. diminuta* adults and cysticercoids, which helps not only to understand specific adaptations to parasitism and invasion, but may also provide the data necessary for planning experiments related to new drug targets, vaccines, or diagnostics.

We used 1-DE for sample separation and were able to analyse all proteins present in a complex mixture of proteins (e.g., from cells, subcellular fractions, and surface proteins). In two-dimensional gel electrophoresis, some proteins could be missed or even lost, as alkaline or hydrophobic proteins (or both) remain unanalyzed since SDS cannot be used for protein solubilisation due to its charged character. Additionally, we have performed in-liquid digestion analyses to confirm the GeLC-MS/MS analyses and select proteins dominant in each of the developmental stages. Both proteomic approaches provide us with different numbers of identifications, however, it seems to be typical that in-solution digestions does not always provide proteome coverage as high as that of the in-gel approach (Liebler and Ham, 2009; Choksawangkarn et al., 2012).

Laschuk et al. (2011) applied proteomic approaches to produce the first proteomic profile of non-taeniid *M. corti* tetrathyridia that were induced to undergo strobilation. Identified proteins were basic metabolic enzymes and proteins associated with the cytoskeleton, chaperone systems, protein synthesis, and turnover. We observed similar protein families in both developmental stages of *H. diminuta*. Some of these key proteins are observed only in the cysticercoid or exclusively in the adult stage; we will discuss their possible role in cestode biology in more detail.

In the paper by Kim et al. (2009), differential protein expression of three different stages of *S. erinacei* (plerocercoid metacestode, pre-strobilated juveniles, and adults) was compared. They observed differences in expression of energy-metabolism enzymes. We have shown that among proteins directly involved in carbohydrate energy metabolism, isocitrate dehydrogenase is specifically expressed only in the cysticercoid stage of *H. diminuta*, whereas triosephosphate isomerase was expressed in both stages. Higher metabolic activity of the cysticercoid may be associated with mechanisms involved in successful infection and with increased metabolic levels during strobilation, which occurs at the beginning of invasion.

Our recent study showed that adult *H. diminuta* excretory-secretory products contain immunogenic proteins, among them key molecules engaged in host-parasite interactions (Bien et al., 2016). Immunomodulatory properties and the complexity of host-parasite interactions were confirmed most recently by Zawistowska-Deniziak et al. (2017), who analyzed human macrophage polarization by *H. diminuta*. In the present study, we identified potential antigenic proteins specific for the cysticercoid and adult stages; however, a number of proteins known as antigens were also identified in both stages. The antigenic proteins of cysticercoids are the first proteins to cause an immunological reaction in a vertebrate host after infection and are therefore the most interesting for diagnostic applications. The presence of these proteins also in the adult stage guarantees that antibodies will be present during the entire course of infection. These data provide information regarding which proteins are the most promising targets for diagnostics or drug development and which proteins could be considered as key molecules in host-parasite interactions (Table 1). The other identified proteins are involved in a wide range of biological functions. Kim et al. (2009) observed that thioredoxin family proteins are highly expressed only in pre-strobilated juvenile worms of *S. erinacei*. This protein plays important roles in protection from reactive oxygen species produced by host inflammatory cells. The presence of thioredoxin in both consecutive developmental stages of *H. diminuta* indicates a common mechanism of host immune evasion.

Both developmental stages expressed 14-3-3 protein. This protein is an important vaccine candidate and is commonly observed as an immunogenic protein (Santivañez et al., 2010; Ludolf et al., 2014). This protein is considered to be a cytosolic molecule; however, it is also classified as a surface protein in oncospheres of *T. solium* (Santivañez et al., 2010) and is present in the excretory-secretory products of flukes. As reviewed by Siles-Lucas et al. (2008), 14-3-3 plays a critical role in eukaryotic cell signaling events that are involved in cell cycle progression, transcriptional alterations in response to environmental stimuli, and in apoptosis. This highly conserved protein is of fundamental importance in the biology of eukaryotic organisms and for helminths may be involved in parasite proliferation and survival.

Similar to Cui et al. (2013), we identified proteins considered as vaccine candidates. These include tegumental membrane vaccine protein enolase, calpain, and glyceraldehyde-3-phosphate dehydrogenase (GAPDH) and HSP. These molecules were identified in both cysticercoid and adult *H. diminuta*. Moreover, we also found several other potential immunomodulators related to infection and pathogenesis by infectious agents (Tables 1, 2). We know from studies on other parasitic organisms that these immunomodulators are involved in host-parasite interactions. In this regard, selected proteins of medical importance present throughout the cestode life cycle are of particular interest.

Cysticercoid and adult *H. diminuta* share at least 104 proteins in gels and 21 proteins in liquid digestion, the majority of which are involved in metabolic processes and host-parasite interaction. These important common features observed in the protein profiles of two consecutive developmental stages of *H. diminuta* may support Conn's statements (Conn, 2000, 2005). Conn posits that the true metamorphosis of hexacanths into stages that are morphologically like immature adults makes the metacestodes of cestodes ontogenetically similar to “juveniles” of other animal phyla, including those that have multiple larval stages and other phyla that develop directly from embryo through juvenile to adult without a larval stage.

Despite the remarkable similarities between the proteins in cysticercoid and adult *Hymenolepis*, the differences are evident and most probably related to the various functions these proteins play during the cestode life cycle. From our gels, we observed that adult parasites possess 86 stage-specific proteins, whereas 135 proteins are cysticercoid specific. From an experimental parasitology perspective, these molecules are key to understanding the complex mechanisms involved in host-parasite interactions.

### Selected proteins observed only in the cysticercoid

One of the cysticercoid-specific proteins is 78-kDa glucose-regulated protein (GRP78). GRP78 is an immunoglobulin heavy chain binding protein and a member of hsp70 protein family (Munro and Pelham, 1986). Yun et al. (2004) suggest that GRP78 functions as a molecular chaperone in adapting parasites to the new host environment. In *E. granulosus* and *E. multilocularis* infections, EM-GRP78 (*E. multilocularis* GRP78) is an immunodominant antigen (Mühlschlegel et al., 1995). In *Hymenolepis* cysticercoids, the protein may also be of importance in host-parasite interactions. The presence of GRP 78 was confirmed in our recent studies focused on the identification of immunogenic proteins in *H. diminuta* cysticercoids (Sulima et al., 2017).

The antigenic and immunomodulatory properties of calcineurin and calreticulin are known from a study on other helminths (Cui et al., 2013). These two immunomodulatory proteins were observed only in the cysticercoid stage samples from ingel analyses. This finding was not confirmed by 2D-immunoblotting studies of the cysticercoid proteome (Sulima et al., 2017). However, it is known that calcineurin may be engaged in the mechanisms of host invasion, as this molecule performs a variety of functions in immune response to infection and as such may influence the process of antigen presentation (Lee et al., 2007). Calreticulin was shown to be secreted by tissue-phase intestinal larvae of a nematode parasite, which can induce Th2 differentiation (Rzepecka et al., 2008).

The presence of antigens and immunomodulatory proteins observed only in the cysticercoid may indicate specialized mechanisms adapted by metacestode stages to evade the host immune system and to establish a successful infection.

Proteins involved in the following molecular functions were distinguished only in the cysticercoid-associated proteome: signal transducer activity, receptor activity, phospholipid binding, and enzyme regulator activity. These functions are associated with processes specific for cysticercoids such as cell adhesion, signal transduction, and stage-specific cellular metabolic processes, which all allow cysticercoid stages to establish a successful infection and response to oxidative stress.

### Selected proteins observed only in the adult stage

Mature *Hymenolepis* must survive in the host intestine to produce progeny. To do so, it must evade the immune response while not causing excessive harm to the host. As discussed above, adult tapeworms can be thought of as a further developed and fully differentiated juvenile stage (cysticercoid). However, the adult parasite, as a sexually mature stage of the cestode life cycle, possesses its own molecular characteristics and adaptations. One of these proteins is the basement membrane-specific heparan sulfate protein, known as a potential antigen in *E. granulosus* hydatid cysts (Pagnozzi et al., 2014). The basement membrane-specific heparan sulfate protein was observed in the immunoreactive bands of *H. diminuta* excretory-secretory products (Bien et al., 2016). This confirms its antigenicity; however, its role in the cestode life cycle remains unclear.

Fumarases or fumarate hydratases are ubiquitous and participate in canalisation of the stereospecific reversible hydration of fumarate to l-malate during the citric acid cycle. Among parasitic worms, this enzyme is very well characterized for the nematode *Ascaris suum*, which, similarly to *H. diminuta*, is an intestinal parasite living in anaerobic conditions. It was shown that in the absence of the citric acid cycle in the parasitic roundworm *A. suum*, the enzyme has been implicated in dismutation of malate in the ascarid mitochondria (Kulkarni et al., 2004). Fumarase occupies the penultimate step prior to succinate formation for fatty acid synthesis in the generation of ATP and seems to be essential for optimal malic enzyme activity. Kulkarni et al. (2004) underscored the usefulness of fumarase as an important target for designing effective drugs for ascariasis. According to their conclusion, since malic enzyme and fumarase share a common fumarate binding site and a malate site, such agents would provide double benefits for chemotherapy. The absence of fumarase in the cysticercoid stage of *H. diminuta* may indicate metabolic diversity, metabolic switch-over or both. Simultaneously, the presence of fumarase in the adult stage makes this enzyme a possible drug target for anthelminthic therapy in the definitive hosts. The enzymes involved in the catabolism of malate (fumarate reductase, NADH oxidase, malic enzyme, succinate dehydrogenase and fumarase and NADPH:NAD transhydrogenase) were studied in *H. diminuta* by Wani and Srivastava (1994). They showed that the cation K+ had no effect on any enzyme whereas Ca2+ and Mg2+ showed an increase or decrease of varying degrees of different enzyme activities. Their research show that compounds synthesized by them may possess anthelmintic properties, strongly inhibited the above enzymes except malic enzyme. The Sj-Ts4 protein, which may be a potential vaccine target for schistosomiasis (Zhou et al., 2002), also needs further investigation.

Not surprisingly, the oncosphere proteins were found to be characteristic for the *H. diminuta* adult stage, as fully formed oncospheres are present in the gravid proglottids. These include oncosphere protein Tso22a (troponin) and oncosphere antigen. Several studies indicate the antigenic potential of taeniid oncospheres (Tsai et al., 2013; Zhang et al., 2014). Taeniid and *H. diminuta* life cycles are distinct; while in taeniid species the egg contains invasive hexacanths able to infect a vertebrate host, in *H. diminuta* eggs the oncosphere is invasive for the invertebrate intermediate host. We do not think that the oncosphere proteins of *H. diminuta* play an important role in parasite interactions with its vertebrate host. Oncosphere proteins of *H. diminuta*, however, may have diagnostics potential and should be evaluated in future studies. Furthermore, oncosphere proteins of *H. diminuta* may be similar to those in the hexacanths of *H. nana*, which do have the ability to invade the tissues of vertebrate definitive hosts. Similar to our study of cysticercoids and adults, the study by Cui et al. (2013) actually compared hydatid metacestodes with adults and did not distinguish adult from larval oncosphere/hexacanth proteins. Similarly, Kim et al. (2009) compared juvenile and adult *S. erinacei* without distinction of the oncospheres contained within the adult. “Adult” cestode tissue in the definitive host actually contains oncosphere/hexacanth larval material from the next genetically distinct generation. Cysticercoids and hydatids (all metacestodes) are different ontogenic stages than the adult but are all part of the same genetic generation. Conversely, oncosphere/hexacanth larval stages within the gravid uterus of the adult are not only a distinct developmental stage, but an entirely new generation, genetically distinct from the cysticercoid and adult that preceded them. This is likely to affect some aspects of their proteomics.

Several differences in the presence of structural proteins were observed between the cysticercoid and adult *H. diminuta*. Predominantly, these differences are related to various isoforms of the proteins, such as actins. In light of the fact that the cysticercoid may be considered as a juvenile stage of tapeworms, we did not expect evident differences in the presence of proteins engaged in the cytoskeleton and musculature between these two stages. Nevertheless, we observed potentially adult-specific proteins related to tegument and musculature, such as myoferlin, myophilin, titin, and troponin I. The possible stage specificity of the aforementioned proteins to adult *H. diminuta* may be related to its active motility and movement within the host intestine and nutrient uptake through the tegument. Alternatively, the musculature of the oncospheres present in the gravid adult uterus could be a source of some proteins identified here. Serological screening of the adult *S. mansoni* proteome identified troponin as one of the predominant antigens recovered from the total protein extract but not present among tegumental proteins (Ludolf et al., 2014). Despite its antigenic properties and presence in cestodes, troponin does not possess sufficient diagnostic validity. Troponin was initially suggested as a promising candidate for diagnostics of taeniasis; however, careful analysis with the use of sera from cysticercosis-positive and negative patients showed disappointing results (Mayta et al., 2009). Since cytoskeletal proteins (including troponin) may show antigenic features, its immunomodulatory functions in adult cestodes must be considered.

Titin, the largest known natural protein, is muscle associated and important in the mechanism of muscle contraction. We previously identified titin in adult-stage excretory-secretory products (Bien et al., 2016). Immunoblot analysis suggests its antigenic potential and possible role in immunomodulation. Titin was also found among the most abundant excretory-secretory proteins of *S. japonicum* (Liu et al., 2009) and was suggested as a molecule used by schistosomes in a species-specific manner for host immune evasion. The present results on *H. diminuta* and the previous evidence for the existence of titin among excretory-secretory products suggest its role as an immunomodulator not only in schistosomes (as suggested by Liu et al. 2009) but also in adult cestodes.

Our findings indicating proteins related only to the adult stage may be supported by recently published analyses of the antigenic proteins of *H. diminuta* cysticercoids (Sulima et al., 2017), in which all of the aforementioned proteins are absent. Also our previous data on the immunogenic properties of the adult *H. diminuta* excretory-secretory products show the presence of basement membrane-specific heparan sulfate protein, titin and myoferlin (Bien et al., 2016). These data may suggest the presence of stage-specific mechanisms and molecules involved in adult tapeworm survival in the host.

## Conclusions

We profiled *H. diminuta* cysticercoid and adult proteins and provided data on their molecular functions. Our results should facilitate understanding of the complexity of the cestode life cycle, survival strategies, adaptive features, and host-parasite interactions in both developmental stages. Comparative proteomic approaches indicated a number of important similarities and pointed out potential differences between the cysticercoid and adult stages. In *H. diminuta*, both proteins noted only in one stage and those shared by both are engaged in the mechanisms crucial for parasite invasion, establishing infection and escaping from host immune defenses. The differences in the protein patterns between consecutive developmental stages of *H. diminuta* may reflect specific strategies and adaptation mechanisms used by this organism in changing conditions and during the life cycle. Since the process of host invasion and mechanisms involved in host-parasite interactions are complex and difficult to understand, more research on specific pathways and particular proteins are needed.

## Author contributions

DM: Supervised the work and all proteomic features and helped draft the manuscript; AN, AS, DM, JB, and KS: Conceived and designed the experiments; DM and AS: Acquired the material, analyzed the data and drafted the manuscript; AS, JB, and DM: Conceived and performed the immunological study with rats; DM and JB: Supported AS in all laboratory analyses; KS supported AS and DM in all mass spectrometry data analyses. AN, DC, JB, KS and RS: Participated in the writing and final editing of the manuscript; KS and AN: Participated in the planning of the study; All authors were actively involved in preparing the manuscript with the responsible author. All authors read and approved the final version of the manuscript.

### Conflict of interest statement

The authors declare that the research was conducted in the absence of any commercial or financial relationships that could be construed as a potential conflict of interest.
