# Supplementary material for: Comparative Proteomic Analysis of Hymenolepis diminuta Cysticercoid and Adult Stages
Source: Front Microbiol. 2018 Jan 15;8:2672. doi: 10.3389/fmicb.2017.02672 (PMC5775281; doi:10.3389/fmicb.2017.02672)
Supplement: Supplementary file 1 [file Table1.DOCX]

**Supplementary Table 1. Results of the LC-MS/MS analysis of selected gel fragments. Proteins identified for Hymenolepis diminuta cysticercoid (C) and adult tapeworm (A). Common proteins are marked with grey color.**

| Protein name and species | Presence | | Band no. | | Accession | Score | | Mass | Matches | | Pep(siq) | | Sequences | | Coverage (%) | |
| --- | --- | --- | --- | --- | --- | --- | --- | --- | --- | --- | --- | --- | --- | --- | --- | --- |
|  | **A** | **C** | **A** | **C** |  | **A** | **C** |  | **A** | **C** | **A** | **C** | **A** | **C** | **A** | **C** |
| 1 pyrroline 5 carboxylate dehydrogenase [Hymenolepis microstoma] | - | + | - | 19 | CDS27592.1 | - | 54 | 62672 | - | 2 | - | 2 | - | 2 | - | 3 |
| 14-3-3 protein [Echinococcus granulosus] | + | + | 38 | 30 | ACP21241.1 | 101 | 157 | 28139 | 2 | 5 | 2 | 5 | 2 | 3 | 7 | 8 |
| 14-3-3 protein beta:alpha [Hymenolepis microstoma] | + | + | 40 | 31 | CDS26348.1 | 92 | 153 | 27984 | 3 | 4 | 3 | 4 | 3 | 3 | 7 | 12 |
| 14-3-3 protein epsilon [Hymenolepis microstoma] | + | + | 37 | 30 | CDS28921.1 | 671 | 260 | 29049 | 10 | 8 | 10 | 8 | 7 | 4 | 34 | 13 |
| 14-3-3 protein homolog 2 | - | + | - | 30 | Q8MM75.1 | - | 100 | 27988 | - | 4 | - | 4 | - | 2 | - | 6 |
| 14-3-3 protein zeta: delta [Hymenolepis microstoma] | + | + | 38 | 30 | CDS31680.1 | 332 | 179 | 29663 | 7 | 8 | 7 | 8 | 5 | 3 | 20 | 10 |
| 26s protease regulatory subunit 6a [Hymenolepis microstoma] | - | + | - | 21 | CDS22733.1 | - | 136 | 45523 | - | 7 | - | 7 | - | 4 | - | 5 |
| 26S protease regulatory subunit 8 [Echinococcus multilocularis] | - | + | - | 22 | CDS22728.1 | - | 462 | 48575 | - | 7 | - | 7 | - | 2 | - | 5 |
| 26s protease regulatory subunit s10b [Hymenolepis microstoma] | - | + | - | 23 | CDS27184.1 | - | 87 | 44653 | - | 2 | - | 2 | - | 2 | - | 5 |
| 26S proteasome non ATPase regulatory subunit 12 [Echinococcus granulosus] | - | + | - | 21 | CDS18941.1 | - | 51 | 51985 | - | 2 | - | 2 | - | 2 | - | 3 |
| 26S proteasome non ATPase regulatory subunit 7 [Hymenolepis microstoma] | - | + | - | 25 | CDS27146.1 | - | 91 | 37528 | - | 2 | - | 2 | - | 2 | - | 5 |
| 26S proteasome regulatory subunit N11 [Echinococcus multilocularis] | - | + | - | 28 | CDS22512.1 | - | 94 | 35096 | - | 2 | - | 2 | - | 2 | - | 6 |
| 26S proteasome regulatory subunit S3 [Hymenolepis microstoma] | - | + | - | 20 | CDS24591.1 | - | 78 | 63992 | - | 2 | - | 2 | - | 2 | - | 3 |
| 3 oxoacyl acyl carrier protein reductase [Hymenolepis microstoma] | + | + | 41 | 32 | CDS30447.1 | 617 | 97 | 27863 | 9 | 2 | 9 | 2 | 5 | 2 | 23 | 8 |
| 40S ribosomal protein [Echinococcus granulosus] | - | + | - | 27 | CDS23509.1 | - | 114 | 30493 | - | 3 | - | 3 | - | 1 | - | 2 |
| 40S ribosomal protein S11 [Hymenolepis microstoma] | - | + | - | 41 | CDS27598.1 | - | 73 | 17084 | - | 3 | - | 3 | - | 1 | - | 4 |
| 40S ribosomal protein S13 [Hymenolepis microstoma] | - | + | - | 40 | CDS29777.1 | - | 82 | 17373 | - | 4 | - | 4 | - | 2 | - | 12 |
| 40S ribosomal protein S14 [Echinococcus granulosus] | + | - | 49 | - | EUB62097.1 | 290 | - | 16494 | 4 | - | 4 | - | 2 | - | 15 | - |
| 40S ribosomal protein S16 [Hymenolepis microstoma] | - | + | - | 41 | CDS34693.1 | - | 76 | 16266 | - | 3 | - | 3 | - | 3 | - | 16 |
| 40S ribosomal protein S17 [Hymenolepis microstoma] | - | + | - | 43 | CDS31932.1 | - | 197 | 13705 | - | 4 | - | 4 | - | 2 | - | 15 |
| 40S ribosomal protein S18 [Echinococcus granulosus] | - | + | - | 40 | CDS42101.1 | - | 311 | 17360 | - | 8 | - | 8 | - | 4 | - | 22 |
| 40S ribosomal protein S2 [Hymenolepis microstoma] | + | + | 39 | 31 | CDS29117.1 | 273 | 346 | 28470 | 6 | 9 | 6 | 9 | 4 | 4 | 16 | 13 |
| 40S ribosomal protein S21 [Hymenolepis microstoma] | - | + | - | 47 | CDS29565.1 | - | 69 | 9708 | - | 2 | - | 2 | - | 2 | - | 24 |
| 6 phosphofructokinase [Echinococcus granulosus] | + | - | 16 | - | CDS20605.1 | 234 | - | 89932 | 5 | - | 5 | - | 4 | - | 5 | - |
| 60S ribosomal protein L21 [Hymenolepis microstoma] | + | + | 46 | 39 | CDS18076.1 | 51 | 81 | 18483 | 2 | 2 | 2 | 2 | 1 | 1 | 3 | 8 |
| 60S ribosomal protein L8 [Hymenolepis microstoma] | + | - | 37 | - | CDS32687.1 | 175 | - | 124177 | 3 | - | 3 | - | 1 | - | 1 | - |
| 78 kDa glucose-regulated protein | - | + | - | 1 | Q24895.1 | - | 62 | 72104 | - | 2 | - | 2 | - | 2 | - | 2 |
| Abhydrolase domain containing protein 11 [Hymenolepis microstoma] | + | + | 44 | 13 | CDS33503.1 | 79 | 96 | 86070 | 9 | 10 | 9 | 10 | 1 | 1 | 0 | 0 |
| Aconitate hydratase, mitochondrial [Hymenolepis microstoma] | - | + | - | 13 | CDS25892.1 | - | 126 | 80449 | - | 4 | - | 4 | - | 4 | - | 5 |
| Actin protein 3B [Hymenolepis microstoma] | + | + | 28 | 22 | CDS27806.1 | 120 | 123 | 47865 | 3 | 2 | 3 | 2 | 3 | 1 | 8 | 2 |
| Actin, cytoplasmic type 5 [Hymenolepis microstoma] | + | + | 29 | 1 | CDS21485.1 | 2716 | 300 | 114751 | 56 | 8 | 56 | 8 | 14 | 6 | 38 | 16 |
| Actin, partial [Diphyllobothrium dendriticum] | + | + | 29 | 23 | AAA21482.1 | 3163 | 1673 | 41700 | 65 | 43 | 65 | 43 | 16 | 11 | 51 | 27 |
| Actin-1 | + | + | 28 | 23 | P35432.1 | 593 | 483 | 42143 | 8 | 12 | 8 | 12 | 4 | 6 | 14 | 15 |
| Actin-2 | + | - | 30 | - | P53456.1 | 1275 | - | 42031 | 27 | - | 27 | - | 13 | - | 4 | - |
| Actin-6 | - | + | - | 24 | P53459.1 | - | 357 | 41896 | - | 13 | - | 13 | - | 4 | - | 9 |
| Adenylate kinase [Hymenolepis microstoma] | + | + | 42 | 33 | CDS21649.1 | 276 | 98 | 22427 | 5 | 4 | 5 | 4 | 3 | 1 | 17 | 6 |
| Adenylyltransferase and sulfurtransferase [Hymenolepis microstoma] | - | + | - | 33 | CDS31625.1 | - | 204 | 24851 | - | 5 | - | 5 | - | 2 | - | 10 |
| ADP dependent glucokinase [Hymenolepis microstoma] | + | - | 54 | - | CDS31513.2 | 82 | - | 68385 | 2 | - | 2 | - | 2 | - | 3 | - |
| ADP ribosylation factor 1 [Hymenolepis microstoma] | + | + | 47 | 38 | CDS31611.1 | 110 | 70 | 18989 | 2 | 2 | 2 | 2 | 2 | 2 | 12 | 12 |
| ADP-ribosylation factor [Echinococcus granulosus] | - | + | - | 39 | EUB59848.1 | - | 473 | 20839 | - | 12 | - | 12 | - | 4 | - | 23 |
| Ag5 [Hymenolepis microstoma] | + | - | 46 | - | CDS31472.1 | 154 | - | 55189 | 4 | - | 4 | - | 1 | - | 2 | - |
| Alanine aminotransferase 2 [Hymenolepis microstoma] | + | + | 25 | 20 | CDS31970.1 | 479 | 166 | 52764 | 12 | 4 | 12 | 4 | 8 | 3 | 16 | 5 |
| Aldo keto reductase family 1, member B4 [Hymenolepis microstoma] | + | + | 35 | 26 | CDS26967.1 | 561 | 216 | 34746 | 15 | 6 | 15 | 6 | 7 | 4 | 17 | 12 |
| Alpha actinin, sarcomeric [Hymenolepis microstoma] | - | + | - | 11 | CDS33844.1 | - | 458 | 106047 | - | 12 | - | 11 | - | 7 | - | 6 |
| Alpha-tubulin [Hymenolepis diminuta] | + | - | 25 | - | AAL84895.1 | 1655 | - | 51217 | 31 | - | 31 | - | 14 | - | 33 | - |
| Ankyrin [Echinococcus granulosus] | + | - | 48 | - | CDS20550.1 | 62 | - | 53224 | 2 | - | 2 | - | 1 | - | 1 | - |
| Annexin [Echinococcus granulosus] | + | + | 31 | 25 | CDS23892.1 | 202 | 189 | 39132 | 6 | 3 | 6 | 3 | 4 | 2 | 9 | 5 |
| Apolipoprotein A I binding protein [Hymenolepis microstoma] | - | + | - | 5 | CDS29142.2 | - | 135 | 316268 | - | 4 | - | 4 | - | 4 | - | 1 |
| Arginine: serine rich splicing factor [Hymenolepis microstoma] | - | + | - | 40 | CDS16875.1 | - | 66 | 16658 | - | 2 | - | 2 | - | 2 | - | 12 |
| Arginyl tRNA synthetase, cytoplasmic [Hymenolepis microstoma] | - | + | - | 16 | CDS30937.1 | - | 64 | 74273 | - | 2 | - | 2 | - | 2 | - | 2 |
| Arp2:3 complex 21 kda subunit [Echinococcus granulosus] | - | + | - | 38 | CDS19264.1 | - | 71 | 21227 | - | 2 | - | 2 | - | 1 | - | 5 |
| Arp2:3 complex subunit [Hymenolepis microstoma] | - | + | - | 37 | CDS33319.1 | - | 82 | 20265 | - | 2 | - | 2 | - | 1 | - | 4 |
| Asparaginyl tRNA synthetase, cytoplasmic [Hymenolepis microstoma] | + | - | 21 | - | CDS30938.2 | 201 | - | 65103 | 4 | - | 4 | - | 1 | - | 1 | - |
| Aspartate aminotransferase, mitochondrial [Hymenolepis microstoma] | + | - | 31 | - | CDS33679.1 | 165 | - | 44617 | 4 | - | 4 | - | 4 | - | 10 | - |
| Aspartyl tRNA synthetase, cytoplasmic [Hymenolepis microstoma] | + | + | 24 | 19 | CDS30263.1 | 137 | 115 | 58980 | 2 | 2 | 2 | 2 | 2 | 2 | 4 | 4 |
| ATP binding cassette sub family G [Hymenolepis microstoma] | - | + | - | 24 | CDS29202.1 | - | 229 | 47461 | - | 9 | - | 9 | - | 5 | - | 10 |
| ATP dependent RNA helicase Ddx1 [Hymenolepis microstoma] | - | + | - | 26 | CDS29449.1 | - | 78 | 36189 | - | 3 | - | 3 | - | 3 | - | 8 |
| ATP dependent RNA helicase DDX31 [Hymenolepis microstoma] | - | + | - | 22 | CDS28632.1 | - | 293 | 48894 | - | 7 | - | 7 | - | 4 | - | 9 |
| ATP dependent rna helicase ddx3y [Echinococcus granulosus] | - | + | - | 16 | EUB56979.1 | - | 85 | 72681 | - | 3 | - | 3 | - | 3 | - | 2 |
| ATP synthase subunit alpha mitochondrial [Echinococcus granulosus] | + | + | 25 | 20 | CDS23514.1 | 538 | 715 | 59663 | 12 | 20 | 12 | 20 | 10 | 7 | 20 | 12 |
| ATP synthase subunit beta, mitochondrial [Hymenolepis microstoma] | + | + | 26 | 21 | CDS31901.2 | 536 | 686 | 56163 | 8 | 19 | 8 | 19 | 5 | 7 | 12 | 11 |
| ATP synthase, subunit d [Hymenolepis microstoma] | - | + | - | 35 | CDS23067.1 | - | 78 | 20327 | - | 2 | - | 2 | - | 2 | - | 9 |
| Basement membrane specific heparan sulfate [Hymenolepis microstoma] | + | - | 2 | - | CDS31901.2 | 382 | - | 899497 | 12 | - | 11 | - | 10 | - | 1 | - |
| Beta 13 n galactosyltransferase [Hymenolepis microstoma] | - | + | - | 23 | CDI96694.2 | - | 61 | 48936 | - | 3 | - | 3 | - | 1 | - | 2 |
| Beta centractin [Hymenolepis microstoma] | + | + | 29 | 24 | CDS32491.1 | 112 | 123 | 42337 | 2 | 4 | 2 | 4 | 1 | 3 | 3 | 8 |
| Beta tubulin [Hymenolepis microstoma] | + | + | 25 | 20 | CDS31534.1 | 2340 | 1341 | 50305 | 42 | 33 | 42 | 33 | 13 | 10 | 12 | 12 |
| Beta-tubulin, partial [Hymenolepis microstoma] | + | + | 25 | 20 | AEO79202.1 | 575 | 704 | 41990 | 13 | 18 | 13 | 18 | 4 | 4 | 34 | 10 |
| Calcineurin B-like protein, partial [Echinococcus granulosus] | - | + | - | 36 | AGE12482.1 | - | 62 | 19034 | - | 4 | - | 4 | - | 1 | - | 4 |
| Calcium binding protein [Echinococcus granulosus] | + | - | 48 | - | CDS23332.1 | 331 | - | 40503 | 6 | - | 6 | - | 4 | - | 12 | - |
| Calcium binding protein calreticulin [Hymenolepis microstoma] | - | + | - | 20 | CDS34484.1 | - | 121 | 48943 | - | 3 | - | 3 | - | 1 | - | 3 |
| Calcyphosin protein [Hymenolepis microstoma] | + | + | 41 | 36 | CDS33882.1 | 162 | 276 | 24132 | 3 | 8 | 3 | 8 | 3 | 3 | 14 | 13 |
| CalModulin family member (cmd 1) [Hymenolepis microstoma] | - | + | - | 40 | CDS28106.1 | - | 61 | 16795 | - | 2 | - | 2 | - | 2 | - | 10 |
| Calpain A [Hymenolepis microstoma] | + | + | 18 | 13 | CDS34356.1 | 162 | 98 | 88988 | 4 | 4 | 4 | 4 | 4 | 2 | 4 | 2 |
| Calponin [Hymenolepis microstoma] | + | - | 45 | - | CDS26392.1 | 175 | - | 47256 | 3 | - | 3 | - | 1 | - | 2 | - |
| Calumenin-B [Echinococcus granulosus] | + | + | 34 | 28 | EUB65028.1 | 207 | 324 | 36814 | 3 | 5 | 3 | 5 | 3 | 3 | 10 | 10 |
| cAMP dependent protein kinase regulatory [Hymenolepis microstoma] | - | + | - | 22 | CDS30262.1 | - | 113 | 42324 | - | 3 | - | 3 | - | 2 | - | 5 |
| Carbonyl reductase 1 [Hymenolepis microstoma] | + | - | 38 | - | CDS33086.1 | 96 | - | 31177 | 3 | - | 3 | - | 2 | - | 6 | - |
| Casein kinase ii subunit alpha [Echinococcus granulosus] | + | - | 21 | - | CDS19284.1 | 319 | - | 53710 | 5 | - | 5 | - | 4 | - | 8 | - |
| Cat eye syndrome critical region protein 5 [Echinococcus granulosus] | + | - | 25 | - | CDS15318.1 | 127 | - | 49845 | 3 | - | 3 | - | 2 | - | 4 | - |
| Chaperonin containing TCP1, subunit 2 (beta) [Hymenolepis microstoma] | - | + | - | 19 | CDS27403.1 | - | 175 | 57534 | - | 3 | - | 3 | - | 1 | - | 2 |
| Chaperonin containing TCP1, subunit 5 (epsilon) [Hymenolepis microstoma] | + | + | 22 | 18 | CDS33651.2 | 129 | 200 | 60321 | 3 | 4 | 3 | 4 | 3 | 4 | 7 | 5 |
| Citrate synthase [Hymenolepis microstoma] | - | + | - | 23 | CDS26683.1 | - | 119 | 44428 | - | 3 | - | 3 | - | 3 | - | 8 |
| Coatomer subunit gamma [Hymenolepis microstoma] | - | + | - | 11 | CDS27345.1 | - | 83 | 101755 | - | 3 | - | 3 | - | 2 | - | 2 |
| Coiled coil domain containing protein 96 [Echinococcus granulosus] | + | - | 53 | - | CDS16293.1 | 67 | - | 55492 | 3 | - | 3 | - | 1 | - | 1 | - |
| Coiled coil helix [Hymenolepis microstoma] | - | + | - | 33 | CDS34043.1 | - | 107 | 22164 | - | 3 | - | 3 | - | 2 | - | 10 |
| Collagen alpha 1(V) chain [Hymenolepis microstoma] | + | + | 3 | 4 | CUU99052.1 | 298 | 146 | 178165 | 4 | 3 | 4 | 3 | 3 | 3 | 2 | 2 |
| Conserved hypothetical protein [Hymenolepis microstoma] | + | - | 10 | - | CDS33096.1 | 86 | - | 80078 | 5 | - | 5 | - | 1 | - | 1 | - |
| Cu, Zn superoxide dismutase [Hymenolepis microstoma] | - | + | - | 41 | CDS31342.1 | - | 144 | 16217 | - | 4 | - | 4 | - | 2 | - | 11 |
| Cysteine desulfurase, mitochondrial [Hymenolepis microstoma] | - | + | - | 23 | CDS34348.1 | - | 59 | 48781 | - | 2 | - | 2 | - | 2 | - | 5 |
| Cytochrome c oxidase subunit Va [Hymenolepis microstoma] | - | + | - | 42 | CDS15227.1 | - | 101 | 18425 | - | 2 | - | 2 | - | 1 | - | 5 |
| Cytochrome c oxidase subunit Vb: COX4 [Hymenolepis microstoma] | - | + | - | 31 | CDS25266.2 | - | 116 | 26089 | - | 2 | - | 2 | - | 2 | - | 5 |
| Cytosolic malate dehydrogenase [Hymenolepis microstoma] | + | + | 34 | 28 | CDS29929.1 | 293 | 94 | 37321 | 10 | 3 | 10 | 3 | 5 | 3 | 13 | 8 |
| Deoxyhypusine hydroxylase:monooxygenase [Hymenolepis microstoma] | + | + | 31 | 24 | CDS31349.1 | 114 | 173 | 39225 | 2 | 3 | 2 | 3 | 2 | 1 | 5 | 3 |
| Dihydrolipoamide dehydrogenase [Hymenolepis microstoma] | + | - | 26 | - | CDS33411.1 | 100 | - | 53836 | 2 | - | 2 | - | 2 | - | 5 | - |
| Dihydrolipoyllysine residue acetyltransferase [Hymenolepis microstoma] | - | + | - | 48 | CDS25294.2 | - | 82 | 10631 | - | 2 | - | 2 | - | 2 | - | 10 |
| Dihydropyrimidinase [Hymenolepis microstoma] | - | + | - | 19 | CDS25739.1 | - | 159 | 65512 | - | 3 | - | 3 | - | 1 | - | 2 |
| Dihydropyrimidine dehydrogenase (NADP+) [Hymenolepis microstoma] | + | - | 11 | - | CDS26708.1 | 61 | - | 120982 | 2 | - | 2 | - | 2 | - | 1 | - |
| Dipeptidyl peptidase 3 [Hymenolepis microstoma] | + | + | 17 | 14 | CUU98717.1 | 205 | 180 | 82288 | 4 | 5 | 4 | 5 | 4 | 4 | 6 | 6 |
| DnaJ subfamily A [Hymenolepis microstoma] | - | + | - | 20 | CDS28557.1 | - | 147 | 45252 | - | 3 | - | 3 | - | 2 | - | 6 |
| Dnl2 protein [Hymenolepis microstoma] | + | - | 55 | - | CDS28557.1 | 133 | - | 10424 | 4 | - | 4 | - | 3 | - | 32 | - |
| Dynein heavy chain [Echinococcus granulosus] | + | - | 44 | - | CDS18210.1 | 62 | - | 478525 | 2 | - | 2 | - | 1 | - | 0 | - |
| Dynein light chain [Echinococcus granulosus] | + | + | 54 | 49 | CDS16711.1 | 104 | 84 | 22510 | 3 | 5 | 3 | 5 | 3 | 3 | 12 | 31 |
| E3 UFM1 protein ligase 1 [Hymenolepis microstoma] | + | + | 24 | 19 | CDS31906.1 | 150 | 222 | 60228 | 4 | 6 | 4 | 6 | 4 | 6 | 8 | 10 |
| EF hand family protein [Hymenolepis microstoma] | + | + | 34 | 28 | CDS30131.1 | 180 | 250 | 37252 | 4 | 8 | 4 | 8 | 4 | 5 | 13 | 11 |
| Elongation factor 1 alpha [Hymenolepis microstoma] | + | - | 27 | - | CDS27990.1 | 294 | - | 101406 | 7 | - | 7 | - | 5 | - | 5 | - |
| Elongation factor 1-a [Hepatoxylon sp.] | + | + | 30 | 29 | AAF13127.1 | 162 | 70 | 28173 | 3 | 2 | 3 | 2 | 1 | 2 | 4 | 7 |
| Elongation factor 2 [Hymenolepis microstoma] | + | - | 14 | - | CDS32105.1 | 199 | - | 95061 | 5 | - | 5 | - | 3 | - | 2 | - |
| Endophilin B1 [Hymenolepis microstoma] | + | + | 40 | 32 | CDS27698.1 | 323 | 166 | 22479 | 9 | 5 | 9 | 5 | 4 | 3 | 11 | 7 |
| Endophilin B2 [Echinococcus granulosus] | - | + | - | 30 | CDS22074.1 | - | 124 | 28904 | - | 2 | - | 2 | - | 1 | - | 3 |
| Enolase [Hymenolepis microstoma] | + | + | 27 | 21 | CDS30005.1 | 1203 | 480 | 48584 | 23 | 13 | 23 | 13 | 10 | 10 | 23 | 20 |
| Enolase, partial [Hymenolepis diminuta] | + | - | 28 | - | AAC47639.1 | 389 | - | 17916 | 5 | - | 5 | - | 3 | - | 23 | - |
| Ester hydrolase C11orf54 [Hymenolepis microstoma] | + | - | 54 | - | CDS27650.1 | 42 | - | 66085 | 2 | - | 2 | - | 1 | - | 2 | - |
| Eukaryotic translation initiation factor 4E [Hymenolepis microstoma] | - | + | - | 35 | CDS31183.1 | - | 54 | 22530 | - | 2 | - | 2 | - | 2 | - | 5 |
| Expressed conserved protein [Echinococcus granulosus] | + | + | 46 | 28 | CDS24072.1 | 121 | 127 | 39603 | 6 | 5 | 6 | 5 | 1 | 1 | 1 | 1 |
| Expressed protein [Hymenolepis microstoma] | + | + | 23 | 18 | CDS27945.1 | 168 | 310 | 59688 | 6 | 10 | 6 | 10 | 6 | 7 | 12 | 12 |
| F actin capping protein subunit beta [Hymenolepis microstoma] | - | + | - | 30 | CDS33771.1 | - | 91 | 30899 | - | 2 | - | 2 | - | 2 | - | 7 |
| Fatty acid binding protein a [Hymenolepis microstoma] | + | - | 51 | - | CDS27704.1 | 489 | - | 15323 | 6 | - | 6 | - | 2 | - | 16 | - |
| Fatty acid binding protein, adipocyte [Hymenolepis microstoma] | + | - | 51 | - | CDS33730.1 | 48 | - | 15258 | 2 | - | 2 | - | 1 | - | 4 | - |
| Fibrillar collagen, partial [Taenia asiatica] | - | + | - | 27 | ABN14935.1 | - | 139 | 19786 | - | 5 | - | 5 | - | 3 | - | 13 |
| Filamin [Echinococcus granulosus] | + | - | 24 | - | CDS23476.1 | 562 | - | 279474 | 9 | - | 9 | - | 5 | - | 2 | - |
| Filamin-A [Echinococcus granulosus] | + | - | 8 | - | EUB63537.1 | 165 | - | 308295 | 6 | - | 6 | - | 5 | - | 1 | - |
| Fk506 binding protein [Hymenolepis microstoma] | - | + | - | 46 | CDS26820.1 | - | 144 | 11702 | - | 2 | - | 2 | - | 1 | - | 11 |
| Four and a half LIM domains protein 2 [Hymenolepis microstoma] | + | - | 37 | - | CDS25251.1 | 372 | - | 65757 | 6 | - | 6 | - | 5 | - | 11 | - |
| Fructose 1,6 bisphosphate aldolase [Hymenolepis microstoma] | + | + | 31 | 25 | CDS26447.1 | 743 | 320 | 39942 | 15 | 6 | 15 | 6 | 6 | 6 | 17 | 15 |
| Fumarate hydratase class I [Echinococcus granulosus] | + | - | 23 | - | CDS20347.1 | 152 | - | 62092 | 3 | - | 3 | - | 3 | - | 7 | - |
| G protein gamma subunit [Hymenolepis microstoma] | - | + | - | 49 | CDS26950.2 | - | 80 | 92760 | - | 4 | - | 4 | - | 2 | - | 1 |
| Gag pol polyprotein [Hymenolepis microstoma] | - | + | - | 36 | CDS27015.1 | - | 38 | 20356 | - | 2 | - | 2 | - | 1 | - | 3 |
| Gelsolin [Echinococcus granulosus] | + | + | 29 | 24 | CDS41196.1 | 126 | 164 | 42226 | 3 | 4 | 3 | 4 | 1 | 1 | 2 | 2 |
| Glucose regulated protein GRP78 [Spirometra erinaceieuropaei] | + | - | 18 | - | AAP84347.1 | 553 | - | 71597 | 10 | - | 10 | - | 5 | - | 7 | - |
| Glutamate dehydrogenase [Hymenolepis microstoma] | + | + | 26 | 21 | CDS29594.1 | 588 | 294 | 58437 | 19 | 10 | 19 | 10 | 5 | 4 | 8 | 5 |
| Glutamate dehydrogenase, mitochondrial [Hymenolepis microstoma] | + | - | 25 | - | CDS25323.1 | 460 | - | 55360 | 14 | - | 14 | - | 7 | - | 10 | - |
| Glutathione S transferase [Hymenolepis microstoma] | + | + | 42 | 34 | CDS31522.1 | 114 | 114 | 15323 | 5 | 4 | 5 | 4 | 2 | 2 | 13 | 7 |
| Glutathione S transferase mu 2 [Hymenolepis microstoma] | - | + | - | 33 | CDS25704.1 | - | 100 | 25905 | - | 2 | - | 2 | - | 1 | - | 4 |
| Glyceraldehyde 3 phosphate dehydrogenase [Hymenolepis microstoma] | + | + | 34 | 27 | CDS31614.1 | 898 | 193 | 36804 | 12 | 4 | 12 | 4 | 6 | 3 | 24 | 8 |
| Glycogen phosphorylase [Hymenolepis microstoma] | + | + | 15 | 12 | CDS19686.1 | 364 | 81 | 97910 | 16 | 4 | 16 | 4 | 9 | 3 | 8 | 2 |
| Glycyl tRNA synthetase [Hymenolepis microstoma] | - | + | - | 15 | CDS31558.1 | - | 67 | 77105 | - | 2 | - | 2 | - | 2 | - | 2 |
| GTP binding nuclear protein Ran [Echinococcus granulosus] | + | - | 41 | - | EUB60932.1 | 77 | - | 24567 | 3 | - | 3 | - | 3 | - | 11 | - |
| Guanine nucleotide binding protein subunit [Hymenolepis microstoma] | - | + | - | 28 | CDS27970.1 | - | 132 | 38078 | - | 3 | - | 3 | - | 2 | - | 6 |
| Gynecophoral canal protein [Hymenolepis microstoma] | + | - | 21 | - | CDS32016.1 | 147 | - | 70529 | 2 | - | 2 | - | 1 | - | 2 | - |
| Heat shock 70kDa protein, partial [Mesocestoides corti] | + | + | 18 | 15 | AAB18390.1 | 495 | 873 | 70925 | 8 | 18 | 8 | 18 | 5 | 10 | 23 | 17 |
| Heat shock protein 60 [Echinococcus multilocularis] | - | + | - | 18 | CDS35950.1 | - | 503 | 60984 | - | 13 | - | 13 | - | 9 | - | 16 |
| Heat shock protein 70 [Hymenolepis microstoma] | + | + | 19 | 15 | CDS28178.2 | 2235 | 734 | 70972 | 38 | 17 | 38 | 10 | 16 | 10 | 24 | 16 |
| Heat shock protein 71 kDa protein [Hymenolepis microstoma] | + | - | 15 | - | CDS28182.1 | 741 | - | 84418 | 17 | - | 17 | - | 13 | - | 17 | - |
| Heat shock protein 75 kDa, mitochondrial [Hymenolepis microstoma] | - | + | - | 15 | CDS32117.1 | - | 128 | 79347 | - | 3 | - | 3 | - | 3 | - | 4 |
| Heat Shock protein family member (hsp 3) [Hymenolepis microstoma] | + | + | 18 | 15 | CDS27455.1 | 553 | 559 | 72678 | 10 | 12 | 10 | 12 | 10 | 6 | 9 | 8 |
| Heat shock protein HSP 90-alpha [Echinococcus granulosus] | + | + | 15 | 12 | EUB54574.1 | 787 | 748 | 84943 | 25 | 17 | 25 | 17 | 15 | 8 | 18 | 10 |
| Hexaprenyldihydroxybenzoate methyltransferase [Hymenolepis microstoma] | + | - | 52 | - | CDS31419.1 | 56 | - | 25106 | 4 | - | 1 | - | 1 | - | 2 | - |
| High mobility group B3 protein [Hymenolepis microstoma] | - | + | - | 31 | CDS32826.2 | - | 107 | 25405 | - | 2 | - | 2 | - | 1 | - | 3 |
| Histone cluster 2, H3c2 [Hymenolepis microstoma] | + | - | 49 | - | CDS27954.1 | 107 | - | 21890 | 5 | - | 5 | - | 3 | - | 11 | - |
| Histone deacetylase [Hymenolepis microstoma] | + | - | 45 | - | CDS31136.1 | 79 | - | 56305 | 3 | - | 3 | - | 1 | - | 1 | - |
| Histone H2A [Echinococcus multilocularis] | + | + | 50 | 45 | CDS23690.1 | 139 | 109 | 13762 | 3 | 8 | 3 | 8 | 1 | 2 | 6 | 12 |
| Histone H2B [Echinococcus multilocularis] | + | + | 50 | 43 | CDS36085.1 | 280 | 307 | 16516 | 7 | 8 | 7 | 8 | 5 | 4 | 34 | 24 |
| histone [*Echinococcus granulosus*] | - | + | - | 41 | CDS15495.1 | - | 98 | 34907 | - | 4 | - | 4 | - | 3 | - | 7 |
| Hydrocephalus inducing protein [Hymenolepis microstoma] | + | + | 12 | 46 | CDS29434.1 | 55 | 49 | 471081 | 2 | 2 | 2 | 2 | 1 | 1 | 0 | 0 |
| Hydrophobic ligand binding protein [Hymenolepis diminuta] | + | - | 53 | - | AAG09785.1 | 556 | - | 8678 | 16 | - | 16 | - | 6 | - | 49 | - |
| Hypothetical protein EGR_01408 [Echinococcus granulosus] | + | - | 54 | - | EUB63785.1 | 74 | - | 120507 | 5 | - | 5 | - | 1 | - | 0 | - |
| Hypothetical protein EGR_02301 [Echinococcus granulosus] | + | - | 55 | - | EUB62860.1 | 55 | - | 40261 | 3 | - | 3 | - | 1 | - | 2 | - |
| Hypothetical protein EGR_05043 [Echinococcus granulosus] | + | - | 30 | - | EUB60045.1 | 243 | - | 39940 | 6 | - | 5 | - | 5 | - | 13 | - |
| Hypothetical protein EGR_08031 [Echinococcus granulosus] | + | - | 43 | - | EUB57083.1 | 66 | - | 19067 | 2 | - | 2 | - | 1 | - | 7 | - |
| Hypothetical protein EgrG_000888000 [Echinococcus granulosus] | + | + | 44 | 17 | CDS16456.1 | 81 | 54 | 68364 | 5 | 4 | 5 | 4 | 1 | 1 | 1 | 1 |
| Hypothetical protein HmN_000464900 [Hymenolepis microstoma] | + | + | 6 | 48 | CDS32815.2 | 90 | 103 | 387041 | 9 | 11 | 9 | 11 | 1 | 1 | 0 | 0 |
| Hypothetical protein HmN_000500900 [Hymenolepis microstoma] | + | - | 44 | - | CDS33271.1 | 54 | - | 19153 | 6 | - | 6 | - | 1 | - | 7 | - |
| Importin 5 [Hymenolepis microstoma] | + | + | 10 | 9 | CDS33564.1 | 77 | 46 | 128085 | 2 | 2 | 2 | 2 | 2 | 2 | 1 | 1 |
| Innexin unc 9 [Hymenolepis microstoma] | + | - | 28 | - | CDS30393.1 | 206 | - | 57313 | 5 | - | 5 | - | 4 | - | 10 | - |
| Inorganic pyrophosphatase [Hymenolepis microstoma] | + | - | 35 | - | CDS27386.1 | 113 | - | 32642 | 3 | - | 3 | - | 2 | - | 7 | - |
| Inositol monophosphatase 1 [Echinococcus granulosus] | - | + | - | 31 | CDS19226.1 | - | 134 | 29481 | - | 4 | - | 4 | - | 3 | - | 7 |
| Iron dependent peroxidase [Hymenolepis microstoma] | - | + | - | 27 | CDS33687.1 | - | 75 | 51222 | - | 2 | - | 2 | - | 1 | - | 2 |
| Iroquois [Hymenolepis microstoma] | - | + | - | 31 | CDS32756.1 | - | 112 | 28148 | - | 2 | - | 2 | - | 2 | - | 8 |
| Isocitrate dehydrogenase (NAD) subunit [Hymenolepis microstoma] | - | + | - | 25 | CDS29409.1 | - | 90 | 39877 | - | 3 | - | 3 | - | 2 | - | 4 |
| Isoleucyl tRNA synthetase, cytoplasmic [Hymenolepis microstoma] | - | + | - | 10 | CDS27818.1 | - | 53 | 145673 | - | 3 | - | 3 | - | 3 | - | 1 |
| Kinesin protein kif3b [Hymenolepis microstoma] | - | + | - | 11 | CDS28954.1 | - | 80 | 104299 | - | 3 | - | 3 | - | 3 | - | 2 |
| L lactate dehydrogenase B chain [Echinococcus granulosus] | + | + | 34 | 27 | CDS32958.1 | 77 | 83 | 49664 | 3 | 3 | 3 | 3 | 3 | 1 | 5 | 1 |
| Lactate dehydrogease [Spirometra erinaceieuropaei] | + | - | 33 | - | ADK62519.1 | 115 | - | 36462 | 5 | - | 5 | - | 2 | - | 3 | - |
| Lactate dehydrogenase A [Hymenolepis microstoma] | + | - | 33 | - | CDS26883.1 | 173 | - | 36385 | 7 | - | 7 | - | 4 | - | 7 | - |
| Lamin [Hymenolepis microstoma] | + | + | 20 | 16 | CDS25469.1 | 97 | 68 | 66837 | 3 | 3 | 3 | 3 | 3 | 3 | 3 | 4 |
| Lamin dm0 [Hymenolepis microstoma] | - | + | - | 21 | CDS25470.1 | - | 103 | 49810 | - | 4 | - | 4 | - | 4 | - | 7 |
| Large subunit ribosomal protein l7e [Echinococcus granulosus] | + | - | 37 | - | CDS20544.1 | 90 | - | 37884 | 3 | - | 3 | - | 3 | - | 7 | - |
| MAF protein [Hymenolepis microstoma] | - | + | - | 33 | CDS32586.1 | - | 84 | 22790 | - | 2 | - | 2 | - | 1 | - | 5 |
| Major egg antigen (p40) [Hymenolepis microstoma] | - | + | - | 25 | CDS31595.1 | - | 220 | 35298 | - | 10 | - | 10 | - | 3 | - | 8 |
| Major egg antigen [Hymenolepis microstoma] | - | + | - | 19 | CDS33705.1 | - | 357 | 52831 | - | 8 | - | 8 | - | 5 | - | 9 |
| Major vault protein [Hymenolepis microstoma] | + | + | 12 | 11 | CDS29747.1 | 255 | 92 | 97866 | 7 | 4 | 7 | 4 | 5 | 3 | 5 | 2 |
| Malate dehydrogenase [Echinococcus multilocularis] | - | + | - | 27 | CDS22680.1 | - | 210 | 35961 | - | 4 | - | 4 | - | 3 | - | 10 |
| Mannose 1 phosphate guanyltransferase beta [Hymenolepis microstoma] | - | + | - | 24 | CDS22572.1 | - | 66 | 40475 | - | 2 | - | 2 | - | 2 | - | 4 |
| Mitochondrial import receptor subunit tom34 [Hymenolepis microstoma] | - | + | - | 20 | CDS31809.1 | - | 125 | 40768 | - | 2 | - | 2 | - | 1 | - | 3 |
| Mitochondrial intermediate peptidase [Hymenolepis microstoma] | - | + | - | 16 | CDS32384.1 | - | 73 | 68815 | - | 2 | - | 2 | - | 2 | - | 3 |
| Mitochondrial processing peptidase beta subunit [Hymenolepis microstoma] | - | + | - | 21 | CDI97367.1 | - | 69 | 53767 | - | 3 | - | 3 | - | 2 | - | 2 |
| Multivalent antigen sj tpi [Echinococcus granulosus] | - | + | - | 11 | CDS20084.1 | - | 47 | 103960 | - | 2 | - | 2 | - | 2 | - | 1 |
| Muscle M line assembly protein unc 89, partial [Hymenolepis microstoma] | + | - | 11 | - | CDS25384.2 | 154 | - | 451092 | 3 | - | 3 | - | 2 | - | 0 | - |
| Myoferlin [Hymenolepis microstoma] | + | - | 4 | - | CDS32028.1 | 171 | - | 235262 | 4 | - | 4 | - | 4 | - | 1 | - |
| Myosin 2 essential light chain [Hymenolepis microstoma] | - | + | - | 43 | CDS28312.1 | - | 88 | 16649 | - | 3 | - | 3 | - | 2 | - | 13 |
| Myosin essential light chain [Hymenolepis microstoma] | + | + | 49 | 41 | CDS33375.1 | 264 | 158 | 20437 | 6 | 5 | 6 | 5 | 4 | 3 | 26 | 16 |
| Myosin heavy chain [Hymenolepis microstoma] | + | + | 5 | 5 | CDS33163.1 | 10224 | 1049 | 224303 | 180 | 30 | 180 | 30 | 75 | 20 | 38 | 10 |
| Myosin heavy chain, non muscle [Hymenolepis microstoma] | + | + | 4 | 5 | CDS19483.1 | 276 | 65 | 232171 | 6 | 3 | 6 | 3 | 6 | 3 | 3 | 0 |
| Myosin regulatory light chain [Taenia asiatica] | + | + | 42 | 33 | ABN14929.1 | 227 | 93 | 19551 | 8 | 3 | 8 | 3 | 4 | 2 | 16 | 11 |
| NADH cytochrome b5 reductase 3 [Echinococcus granulosus] | - | + | - | 29 | CDS16240.1 | - | 56 | 36892 | - | 2 | - | 2 | - | 1 | - | 2 |
| NADP binding domain [Echinococcus granulosus] | - | + | - | 27 | CDS19779.1 | - | 134 | 48981 | - | 6 | - | 6 | - | 3 | - | 7 |
| NADP dependent malic enzyme [Hymenolepis microstoma] | + | + | 22 | 18 | CDS35028.1 | 127 | 210 | 63550 | 3 | 6 | 3 | 6 | 3 | 6 | 3 | 9 |
| Ndr [Hymenolepis microstoma] | + | - | 30 | - | CDS30121.1 | 337 | - | 40995 | 8 | - | 7 | - | 6 | - | 16 | - |
| Negative elongation factor B [Hymenolepis microstoma] | + | + | 8 | 9 | CDS33580.1 | 74 | 67 | 110938 | 5 | 4 | 5 | 4 | 1 | 1 | 0 | 0 |
| Neuronal calcium sensor [Hymenolepis microstoma] | + | + | 23 | 18 | CDS29101.2 | 102 | 236 | 56386 | 2 | 5 | 2 | 5 | 2 | 3 | 2 | 4 |
| Nuclear pore complex protein Nup205 [Hymenolepis microstoma] | + | - | 18 | - | CDS25977.1 | 108 | - | 354920 | 2 | - | 2 | - | 2 | - | 0 | - |
| Nucleolin [Hymenolepis microstoma] | - | + | - | 27 | CDS31961.1 | - | 59 | 37294 | - | 2 | - | 2 | - | 1 | - | 2 |
| Nucleoside diphosphate kinase A [Hymenolepis microstoma] | + | + | 50 | 42 | CDS25930.1 | 221 | 106 | 17298 | 6 | 5 | 6 | 5 | 3 | 3 | 20 | 16 |
| NudC domain containing protein 3 [Echinococcus granulosus] | - | + | - | 1 | CDS19548.1 | - | 64 | 44107 | - | 2 | - | 3 | - | 1 | - | 2 |
| Oncosphere antigen [Echinococcus multilocularis] | + | - | 48 | - | CAD12370.1 | 59 | - | 17205 | 3 | - | 3 | - | 1 | - | 7 | - |
| Oncosphere protein Tso22a [Hymenolepis microstoma] | + | - | 30 | - | CDS28056.1 | 400 | - | 38823 | 12 | - | 12 | - | 7 | - | 17 | - |
| Ornithine aminotransferase [Hymenolepis microstoma] | + | + | 29 | 23 | CDS29109.2 | 139 | 195 | 46104 | 4 | 4 | 4 | 4 | 2 | 2 | 4 | 4 |
| p38-like protein [Echinococcus granulosus] | - | + | - | 23 | ACT21201.1 | - | 46 | 42295 | - | 2 | - | 2 | - | 1 | - | 2 |
| Paramyosin [Hymenolepis microstoma] | + | + | 13 | 11 | CDS32748.2 | 6226 | 997 | 99179 | 125 | 27 | 124 | 27 | 44 | 17 | 42 | 17 |
| Peroxidasin extracellular matrix-associated peroxidase [Hymenolepis microstoma] | + | - | 8 | - | CDS27358.1 | 66 | - | 152617 | 2 | - | 2 | - | 2 | - | 1 | - |
| Peroxiredoxin 2 [Hymenolepis microstoma] | - | + | - | 35 | CDS27844.2 | - | 145 | 21950 | - | 5 | - | 5 | - | 2 | - | 8 |
| PHD and RING finger domain containing protein 1 [Echinococcus granulosus] | - | + | - | 13 | CDS20146.1 | - | 47 | 92146 | - | 3 | - | 3 | - | 1 | - |  |
| Phosphatase 2a inhibitor i2pp2a [Echinococcus granulosus] | - | + | - | 27 | CDS19354.1 | - | 85 | 34339 | - | 2 | - | 2 | - | 1 | - | 2 |
| Phosphoenolpyruvate carboxykinase [Hymenolepis microstoma] | + | + | 20 | 16 | CDS27807.1 | 2404 | 345 | 71158 | 50 | 13 | 50 | 13 | 17 | 7 | 30 | 11 |
| phosphoenolpyruvate carboxykinase, partial [Taenia serialis] | + | - | 20 | - | CBH36501.1 | 1141 | - | 55077 | 23 | - | 23 | - | 7 | - | 17 | - |
| Phosphoglucomutase [Hymenolepis microstoma] | + | - | 21 | - | CDS26093.1 | 205 | - | 127353 | 4 | - | 4 | - | 3 | - | 3 | - |
| Phosphoglycerate kinase 1 [Hymenolepis microstoma] | + | + | 29 | 23 | CDS32456.1 | 645 | 231 | 44562 | 11 | 8 | 11 | 8 | 8 | 4 | 8 | 9 |
| Phosphoglycerate mutase [Hymenolepis microstoma] | + | + | 39 | 31 | CDS15596.1 | 424 | 184 | 28839 | 9 | 5 | 9 | 5 | 5 | 2 | 22 | 7 |
| Phytochelatin synthase [Hymenolepis microstoma] | - | + | - | 34 | CDS31607.1 | - | 89 | 22217 | - | 2 | - | 2 | - | 2 | - | 9 |
| PIK kinase, FAT and Armadillo domain containing protein [Echinococcus multilocularis] | - | + | - | 8 | CDS22626.1 | - | 37 | 509796 | - | 2 | - | 2 | - | 1 | - | 0 |
| Polycomb group ring finger protein 3 [Hymenolepis microstoma] | - | + | - | 37 | CDS28447.1 | - | 58 | 22147 | - | 4 | - | 3 | - | 2 | - | 4 |
| Pre mRNA processing factor 40 B [Hymenolepis microstoma] | + | - | 8 | - | CDS30206.1 | 66 | - | 190520 | 3 | - | 3 | - | 2 | - | 0 | - |
| Presequence protease, mitochondrial [Hymenolepis microstoma] | - | + | - | 10 | CDS34330.2 | - | 73 | 109742 | - | 2 | - | 2 | - | 2 | - | 1 |
| Probable Ras-related protein Rab-4A | - | + | - | 33 | Q9GP33.1 | - | 73 | 24667 | - | 2 | - | 2 | - | 1 | - | 4 |
| Procollagen lysine,2 oxoglutarate 5 dioxygenase [Hymenolepis microstoma] | + | + | 17 | 13 | CDS25586.1 | 84 | 341 | 82804 | 2 | 12 | 2 | 12 | 2 | 4 | 2 | 4 |
| Prohibitin [Hymenolepis microstoma] | + | + | 36 | 29 | CDS33227.1 | 168 | 436 | 30823 | 4 | 11 | 4 | 11 | 4 | 5 | 14 | 16 |
| Proteasome (prosome macropain) subunit beta [Hymenolepis microstoma] | - | + | - | 33 | CDS31636.2 | - | 68 | 22167 | - | 3 | - | 3 | - | 2 | - | 4 |
| Protein disulfide isomerase [Echinococcus granulosus] | + | + | 24 | 19 | CDS24783.1 | 95 | 63 | 59255 | 4 | 3 | 4 | 3 | 3 | 3 | 4 | 2 |
| Protein DJ 1 [Hymenolepis microstoma] | - | + | - | 36 | CDS32327.1 | - | 126 | 19906 | - | 2 | - | 2 | - | 1 | - | 6 |
| Protein phosphatase 1b [Echinococcus granulosus] | - | + | - | 24 | CDS23125.1 | - | 90 | 42115 | - | 2 | - | 2 | - | 1 | - | 2 |
| Pseudouridine metabolizing bifunctional protein [Hymenolepis microstoma] | + | - | 19 | - | CDS29349.1 | 263 | - | 75507 | 5 | - | 5 | - | 4 | - |  | - |
| Putative 14-3-3 protein [Echinococcus granulosus] | - | + | - | 1 | AAX73175.1 | - | 82 | 28213 | - | 2 | - | 2 | - | 2 | - | 6 |
| Putative HSP20 related protein [Echinococcus multilocularis] | - | + | - | 25 | CAD12371.1 | - | 164 | 35690 | - | 7 | - | 7 | - | 2 | - | 5 |
| Putative insulin receptor [Echinococcus multilocularis] | - | + | - | 7 | CAD30260.1 | - | 46 | 195667 | - | 3 | - | 3 | - | 1 | - | 0 |
| Putative major vault protein [Echinococcus multilocularis] | + | + | 12 | 11 | BAN04642.1 | 385 | 81 | 97555 | 7 | 2 | 7 | 2 | 4 | 2 | 4 | 2 |
| Pyruvate dehydrogenase [Echinococcus granulosus] | + | + | 35 | 29 | EUB62991.1 | 150 | 149 | 39544 | 2 | 3 | 2 | 3 | 1 | 1 | 3 | 2 |
| Pyruvate dehydrogenase E1 component subunit [Echinococcus granulosus] | + | + | 31 | 25 | CDS29602.1 | 233 | 87 | 42905 | 6 | 2 | 6 | 2 | 5 | 2 | 9 | 1 |
| Pyruvate kinase [Echinococcus granulosus] | + | + | 22 | 18 | CDS17986.1 | 931 | 109 | 62872 | 20 | 2 | 20 | 2 | 14 | 2 | 26 | 3 |
| Pyruvate kinase isozymes M1:M2 [Hymenolepis microstoma] | + | - | 22 | - | CDS25961.1 | 1126 | - | 114130 | 24 | - | 24 | - | 15 | - | 16 | - |
| RAB6A member RAS oncogene family [Echinococcus granulosus] | - | + | - | 35 | CDS36595.1 | - | 101 | 23777 | - | 3 | - | 3 | - | 2 | - | 8 |
| Radixin [Hymenolepis microstoma] | + | - | 21 | - | CDS33034.2 | 152 | - | 66877 | 2 | - | 2 | - | 2 | - | 3 | - |
| RAS protein rab [Hymenolepis microstoma] | - | + | - | 33 | CDS30879.1 | - | 114 | 27547 | - | 3 | - | 3 | - | 2 | - | 9 |
| Receptor Mediated Endocytosis family member [Hymenolepis microstoma] | - | + | - | 18 | CDS32978.1 | - | 105 | 63666 | - | 5 | - | 5 | - | 5 | - | 6 |
| Receptor type tyrosine protein phosphatase [Echinococcus granulosus] | + | + | 7 | 48 | CDS22104.1 | 121 | 264 | 246580 | 10 | 30 | 9 | 28 | 1 | 1 | 0 | 0 |
| Ribosomal protein [Hymenolepis microstoma] | - | + | - | 38 | CDS28937.1 | - | 132 | 21739 | - | 6 | - | 6 | - | 5 | - | 16 |
| Ribosomal protein L10 [Hymenolepis microstoma] | - | + | - | 33 | CDS29467.1 | - | 98 | 25384 | - | 5 | - | 5 | - | 3 | - | 13 |
| Ribosomal protein L15 [Hymenolepis microstoma] | - | + | - | 34 | CDS31632.1 | - | 87 | 21913 | - | 4 | - | 4 | - | 3 | - | 11 |
| Ribosomal protein L31 [Hymenolepis microstoma] | - | + | - | 40 | CDS32972.1 | - | 61 | 14411 | - | 2 | - | 2 | - | 1 | - | 4 |
| Ribosomal protein l4 [Echinococcus granulosus] | + | - | 31 | - | CDS16186.1 | 131 | - | 51453 | 2 | - | 2 | - | 2 | - | 5 | - |
| Ribosomal protein L9 [Hymenolepis microstoma] | - | + | - | 35 | CDS25972.1 | - | 98 | 20946 | - | 2 | - | 2 | - | 2 | - | 11 |
| Ribosomal protein rpl13a [Hymenolepis microstoma] | - | + | - | 34 | CDS32619.1 | - | 66 | 23785 | - | 3 | - | 3 | - | 3 | - | 12 |
| Ribosomal protein s14 [Hymenolepis microstoma] | - | + | - | 44 | CDS31927.1 | - | 200 | 16712 | - | 5 | - | 5 | - | 2 | - | 15 |
| Ribosomal protein S18 [Taenia asiatica] | - | + | - | 40 | ABN14904.1 | - | 261 | 17704 | - | 7 | - | 7 | - | 4 | - | 23 |
| Ribosomal protein S19e [Hymenolepis microstoma] | - | + | - | 43 | CDS26122.1 | - | 81 | 16499 | - | 3 | - | 3 | - | 2 | - | 10 |
| Ribosomal protein S3 [Hymenolepis microstoma] | + | + | 40 | 31 | CDS26911.1 | 214 | 461 | 26541 | 4 | 13 | 4 | 13 | 4 | 9 | 17 | 34 |
| Ribosomal protein S4 [Hymenolepis microstoma] | + | + | 38 | 30 | CDS25263.2 | 186 | 274 | 30277 | 4 | 9 | 4 | 9 | 3 | 5 | 7 | 11 |
| Rootletin [Echinococcus granulosus] | + | + | 9 | 7 | CDS23873.1 | 62 | 58 | 252063 | 2 | 2 | 2 | 2 | 1 | 1 |  | 0 |
| S adenosylmethionine synthase type 1 [Hymenolepis microstoma] | - | + | - | 21 | CDS28727.1 | - | 100 | 44632 | - | 2 | - | 2 | - | 2 | - | 5 |
| Sarcoplasmic calcium binding protein [Echinococcus granulosus] | + | + | 29 | 24 | EUB60311.1 | 145 | 130 | 45528 | 4 | 5 | 4 | 5 | 3 | 3 | 5 | 5 |
| Sideroflexin 1 [Echinococcus granulosus] | - | + | - | 27 | CDS15926.1 | - | 88 | 36257 | - | 2 | - | 2 | - | 1 | - | 3 |
| SJ TS4 protein [Echinococcus granulosus] | + | - | 39 | - | CDS24565.1 | 121 | - | 38469 | 3 | - | 3 | - | 2 | - | 6 | - |
| Small heat-shock protein [Taenia solium] | - | + | - | 25 | CAD36617.1 | - | 175 | 35640 | - | 8 | - | 8 | - | 2 | - | 7 |
| Small nuclear ribonucleoprotein Sm [Hymenolepis microstoma] | - | + | - | 44 | CDS31982.1 | - | 127 | 13971 | - | 2 | - | 2 | - | 1 | - | 9 |
| SOD [Spirometra erinaceieuropaei] | + | - | 49 | - | AAQ95747.1 | 146 | - | 16168 | 2 | - | 2 | - | 1 | - | 6 | - |
| Sodium/potassium-transporting ATPase subunit alpha | + | + | 6 | 11 | Q6RWA9.1 | 176 | 89 | 113058 | 4 | 2 | 4 | 2 | 4 | 1 | 4 | 0 |
| Sodium:glucose cotransporter 2 [Echinococcus granulosus] | - | + | - | 17 | CDS16808.1 | - | 45 | 71611 | - | 2 | - | 2 | - | 1 | - | 1 |
| Spectrin alpha actinin [Hymenolepis microstoma] | + | + | 11 | 4 | CDS29622.1 | 853 | 1361 | 282888 | 21 | 41 | 20 | 40 | 13 | 30 | 5 | 11 |
| Spectrin beta chain [Hymenolepis microstoma] | + | + | 17 | 4 | CDS28968.2 | 581 | 588 | 272698 | 13 | 18 | 13 | 18 | 11 | 16 | 5 | 6 |
| Spermidine synthase [Echinococcus granulosus] | + | - | 37 | - | CDS19536.1 | 153 | - | 32670 | 2 | - | 2 | - | 1 | - | 4 | - |
| Subfamily T1A non peptidase ue [Echinococcus multilocularis] | + | + | 42 | 33 | CDS22665.1 | 82 | 99 | 24410 | 2 | 2 | 2 | 2 | 2 | 2 | 8 | 9 |
| Succinate dehydrogenase (ubiquinone) [Hymenolepis microstoma] | + | + | 19 | 16 | CDS32637.1 | 566 | 186 | 71769 | 13 | 5 | 13 | 5 | 11 | 3 | 20 | 5 |
| Succinate dehydrogenase (ubiquinone) iron sulfur [Hymenolepis microstoma] | + | - | 38 | - | CDS29964.1 | 110 | - | 41037 | 2 | - | 1 | - | 2 | - | 3 | - |
| Succinyl coenzyme A ligase GDP forming subunit beta [Echinococcus granulosus] | - | + | - | 22 | CDS15772.1 | - | 192 | 47123 | - | 3 | - | 3 | - | 2 | - | 4 |
| Succinyl coenzyme A synthetase alpha subunit [Hymenolepis microstoma] | + | - | 35 | - | CDS28334.1 | 144 | - | 34240 | 3 | - | 3 | - | 2 | - | 9 | - |
| T complex protein 1 subunit alpha [Echinococcus granulosus] | + | + | 22 | 20 | CDS40669.1 | 138 | 126 | 60140 | 4 | 3 | 4 | 3 | 4 | 2 | 8 | 3 |
| T complex protein 1 subunit delta [Hymenolepis microstoma] | + | + | 23 | 18 | CDS26996.1 | 127 | 195 | 58251 | 2 | 5 | 2 | 5 | 2 | 4 | 4 | 8 |
| T complex protein 1 subunit theta [Hymenolepis microstoma] | - | + | - | 19 | CDS39334.1 | - | 320 | 59816 | - | 7 | - | 7 | - | 4 | - | 4 |
| T complex protein 1 subunit zeta [Hymenolepis microstoma] | - | + | - | 18 | CDS29679.2 | - | 244 | 60491 | - | 7 | - | 7 | - | 5 | - | 8 |
| Tegumental protein [Hymenolepis microstoma] | + | + | 46 | 39 | CDS32190.1 | 190 | 56 | 21016 | 2 | 3 | 2 | 3 | 1 | 1 | 8 | 3 |
| Thioredoxin peroxidase [Echinococcus granulosus] | + | + | 43 | 34 | CDS27660.2 | 88 | 216 | 20964 | 2 | 7 | 2 | 7 | 2 | 4 | 9 | 1 |
| Titin [Hymenolepis microstoma] | + | - | 3 | - | CDS32603.2 | 182 | - | 782807 | 6 | - | 6 | - | 4 | - | 0 | - |
| Transitional endoplasmic reticulum atpase [Hymenolepis microstoma] | + | + | 15 | 12 | CDS29022.1 | 116 | 167 | 89034 | 3 | 6 | 3 | 6 | 3 | 4 | 3 | 5 |
| Transketolase [Hymenolepis microstoma] | + | + | 21 | 17 | CDS28549.1 | 383 | 59 | 68433 | 7 | 3 | 7 | 3 | 5 | 2 | 7 | 2 |
| Translation initiation factor 4A-like protein [Echinococcus multilocularis] | - | + | - | 22 | CAC18543.1 | - | 146 | 46591 | - | 4 | - | 4 | - | 3 | - | 7 |
| Translocon associated protein gamma subunit [Hymenolepis microstoma] | - | + | - | 39 | CDS32288.1 | - | 72 | 21397 | - | 2 | - | 2 | - | 1 | - | 5 |
| Triosephosphate isomerase [Hymenolepis microstoma] | + | + | 40 | 31 | CDS29962.1 | 668 | 264 | 27872 | 14 | 7 | 14 | 7 | 8 | 5 | 36 | 22 |
| Tropomyosin | + | + | 34 | 27 | Q95PU1.1 | 759 | 333 | 32967 | 15 | 7 | 15 | 7 | 9 | 6 | 29 | 22 |
| Tropomyosin 1 low molecular weight isoform [Mesocestoides corti] | + | - | 37 | - | ADQ26719.1 | 110 | - | 28578 | 3 | - | 3 | - | 2 | - | 8 | - |
| Tropomyosin 2 [Echinococcus multilocularis] | + | - | 36 | - | CAS07435.1 | 111 | - | 28303 | 2 | - | 2 | - | 2 | - | 8 | - |
| Tropomyosin 2 high molecular weight isoform [Mesocestoides corti] | + | + | 31 | 25 | ADQ26722.1 | 367 | 275 | 31588 | 8 | 7 | 8 | 7 | 8 | 5 | 23 | 18 |
| Tropomyosin 2 low molecular weight isoform [Mesocestoides corti] | - | + | - | 29 | ADQ26721.1 | - | 175 | 23553 | - | 3 | - | 3 | - | 2 | - | 9 |
| Tropomyosin A | + | - | 35 | - | O16127.3 | 236 | - | 32249 | 5 | - | 5 | - | 5 | - | 19 | - |
| Tropomyosin-2 [Echinococcus granulosus] | + | - | 32 | - | EUB56942.1 | 659 | - | 76467 | 12 | - | 12 | - | 7 | - | 7 | - |
| Troponin I [Hymenolepis microstoma] | + | - | 36 | - | CDS29158.1 | 425 | - | 29480 | 6 | - | 6 | - | 4 | - | 18 | - |
| Tubulin [Spirometra erinaceieuropaei] | - | + | - | 21 | AFX72996.1 | - | 199 | 50838 | - | 6 | - | 6 | - | 3 | - | 6 |
| Tubulin alpha 1 chain [Echinococcus granulosus] | + | - | 25 | - | CDI96718.1 | 895 | - | 50971 | 20 | - | 20 | - | 9 | - | 19 | - |
| Tubulin alpha 1C chain [Hymenolepis microstoma] | + | + | 25 | 20 | CDS34320.1 | 957 | 306 | 50847 | 21 | 9 | 21 | 9 | 12 | 6 | 26 | 14 |
| Tubulin alpha chain [Hymenolepis microstoma] | + | - | 25 | - | CDS34223.2 | 983 | - | 35132 | 19 | - | 19 | - | 8 | - | 28 | - |
| Tubulin beta 1 chain [Hymenolepis microstoma] | - | + | - | 20 | CDS31533.1 | - | 601 | 50888 | - | 13 | - | 13 | - | 5 | - | 9 |
| Tubulin beta 2C chain [Hymenolepis microstoma] | + | + | 25 | 20 | CDS26232.1 | 2032 | 1657 | 50360 | 38 | 39 | 38 | 39 | 13 | 11 | 34 | 28 |
| Tubulin beta-2 chain | - | + | - | 23 | Q9NFZ6.1 | - | 127 | 50269 | - | 4 | - | 4 | - | 2 | - | 5 |
| Tumor protein d52 [Hymenolepis microstoma] | - | + | - | 43 | CDS27622.1 | - | 124 | 17273 | - | 2 | - | 2 | - | 1 | - | 8 |
| Type II collagen B [Echinococcus granulosus] | - | + | - | 10 | CDS17835.1 | - | 43 | 154596 | - | 2 | - | 2 | - | 2 | - | 1 |
| U2 small nuclear ribonucleoprotein auxiliary [Hymenolepis microstoma] | + | - | 42 | - | CDS29960.1 | 73 | - | 54451 | 3 | - | 3 | - | 1 | - | 2 | - |
| Ubiquitin (ribosomal protein L40) [Hymenolepis microstoma] | + | + | 49 | 1 | CDS32178.1 | 277 | 339 | 17406 | 6 | 8 | 6 | 8 | 4 | 4 | 26 | 30 |
| Ubiquitin [Echinococcus multilocularis] | + | + | 45 | 49 | AAA29064.1 | 106 | 88 | 8645 | 3 | 3 | 3 | 3 | 2 | 1 | 28 | 5 |
| Ubiquitin conjugating enzyme E2 N [Echinococcus multilocularis] | - | + | - | 40 | CDS26822.1 | - | 66 | 18457 | - | 4 | - | 4 | - | 2 | - | 9 |
| Ubiquitin protein ligase BRE1 [Hymenolepis microstoma] | - | + | - | 16 | CDS33379.1 | - | 83 | 96657 | - | 2 | - | 2 | - | 2 | - | 2 |
| UDP glucose 4 epimerase [Hymenolepis microstoma] | + | - | 33 | - | CDS25480.1 | 199 | - | 39005 | 6 | - | 6 | - | 6 | - | 17 | - |
| Universal stress protein [Hymenolepis microstoma] | - | + | - | 40 | CDS34098.1 | - | 59 | 17857 | - | 2 | - | 2 | - | 1 | - | 6 |
| Vacuolar proton pump subunit E [Echinococcus granulosus] | - | + | - | 31 | CDS43426.1 | - | 83 | 24550 | - | 2 | - | 2 | - | 2 | - | 4 |
| Valyl tRNA synthetase [Hymenolepis microstoma] | - | + | - | 8 | CDS31133.1 | - | 59 | 143231 | - | 3 | - | 3 | - | 2 | - | 1 |
| Vesicle associated membrane protein (vamp) [Hymenolepis microstoma] | - | + | - | 28 | CDS30495.1 | - | 87 | 37424 | - | 2 | - | 2 | - | 1 | - | 3 |
| Viral IAP associated factor [Hymenolepis microstoma] | + | + | 24 | 19 | CDS29466.1 | 347 | 311 | 56018 | 10 | 11 | 10 | 11 | 10 | 6 | 9 | 9 |
