# Supplementary material for: Comparative Proteomic Analysis of Hymenolepis diminuta Cysticercoid and Adult Stages
Source: Front Microbiol. 2018 Jan 15;8:2672. doi: 10.3389/fmicb.2017.02672 (PMC5775281; doi:10.3389/fmicb.2017.02672)
Supplement: Supplementary file 2 [file Table2.DOCX]

**Supplementary Tabel 2. Results of the LC-MS/MS analysis of in-solution digestion samples for proteins identified for cysticercoid (C) and adult tapeworms (A) of Hymenolepis diminuta. Common proteins are marked with grey color.**

| Protein name and species | Presence | | Accession | Score | | Mass | Matches | | Pep(siq) | | Sequences | |
| --- | --- | --- | --- | --- | --- | --- | --- | --- | --- | --- | --- | --- |
|  | **A** | **C** |  | **A** | **C** |  | **A** | **C** | **A** | **C** | **A** | **C** |
| 3 oxoacyl acyl carrier protein reductase [Hymenolepis microstoma] | + | - | gi\|674590667 | 196 | - | 27819 | 4 | - | 4 | - | 2 | - |
| 40S ribosomal protein S18 [Echinococcus granulosus] | + | - | gi\|576698959 | 130 | - | 17348 | 3 | - | 3 | - | 2 | - |
| 78 kDa glucose-regulated protein | - | + | gi\|29336626 | - | 220 | 71815 | - | 4 | - | 4 | - | 4 |
| Actin, cytoplasmic 2 [Echinococcus granulosus] | + | + | gi\|576693212 | 786 | 2077 | 42110 | 18 | 47 | 18 | 47 | 12 | 26 |
| Actin, partial [Diphyllobothrium dendriticum] | + | - | gi\|467215 | 496 | - | 41645 | 14 | - | 14 | - | 12 | - |
| Actin-2 | + | + | gi\|1703110 | 775 | 1299 | 41976 | 19 | 35 | 19 | 35 | 14 | 20 |
| Actin-3 | + | - | gi\|1703116 | 436 | - | 42115 | 12 | - | 12 | - | 10 | - |
| Actin-6 | - | + | gi\|1703124 | - | 684 | 41819 | - | 21 | - | 21 | - | 13 |
| Acyl coenzyme A binding protein [Hymenolepis microstoma] | + | - | gi\|674586889 | 232 | - | 8996 | 3 | - | 3 | - | 3 | - |
| ADP dependent glucokinase [Hymenolepis microstoma] | - | + | gi\|961496727 | - | 151 | 69205 | - | 4 | - | 4 | - | 3 |
| Aldo keto reductase family 1 member B4 [Hymenolepis microstoma] | + | - | gi\|674594288 | 288 | - | 36360 | 9 | - | 9 | - | 6 | - |
| Alpha-tubulin [Hymenolepis diminuta] | + | + | gi\|19073607 | 168 | 295 | 51029 | 2 | 6 | 2 | 6 | 2 | 3 |
| ATP synthase subunit alpha mitochondrial [Hymenolepis microstoma] | - | + | gi\|674592296 | - | 370 | 59655 | - | 6 | - | 6 | - | 6 |
| ATP synthase subunit beta [Echinococcus granulosus] | - | + | gi\|576697808 | - | 173 | 55977 | - | 3 | - | 3 | - | 3 |
| Basement membrane specific heparan sulfate [Hymenolepis microstoma] | + | - | gi\|961498392 | 102 | - | 897113 | 4 | - | 4 | - | 4 | - |
| Beta tubulin [Hymenolepis microstoma] | + | - | gi\|674590829 | 492 | - | 50217 | 8 | - | 8 | - | 7 | - |
| Calumenin-B [Echinococcus granulosus] | + | - | gi\|576701509 | 193 | - | 36803 | 2 | - | 2 | - | 2 | - |
| Clathrin heavy chain [Hymenolepis microstoma] | - | + | gi\|674588966 | - | 234 | 192919 | - | 6 | - | 6 | - | 6 |
| Cu/Zn superoxide dismutase [Hymenolepis microstoma] | - | + | gi\|674589681 | - | 193 | 16173 | - | 3 | - | 3 | - | 2 |
| Cysteine and glycine-rich protein [Echinococcus granulosus] | + | - | gi\|576701105 | 132 | - | 20069 | 3 | - | 3 | - | 3 | - |
| Cytosolic malate dehydrogenase [Taenia solium] | + | - | gi\|323361126 | 139 | - | 36770 | 2 | - | 2 | - | 1 | - |
| Deoxyhypusine hydroxylase [Echinococcus granulosus] | + | - | gi\|576699597 | 105 | - | 96478 | 3 | - | 3 | - | 3 | - |
| Deoxyhypusine hydroxylase:monooxygenase [Hymenolepis microstoma] | + | - | gi\|674589688 | 95 | - | 39170 | 3 | - | 3 | - | 3 | - |
| Dihydrolipoamide dehydrogenase [Hymenolepis microstoma] | + | - | gi\|674587535 | 118 | - | 53659 | 2 | - | 2 | - | 2 | - |
| Dnl2 protein [Hymenolepis microstoma] | + | - | gi\|674592182 | 185 | - | 10402 | 4 | - | 4 | - | 4 | - |
| Dynein light chain 2, cytoplasmic [Echinococcus granulosus] | + | - | gi\|576691996 | 163 | - | 10828 | 4 | - | 4 | - | 4 | - |
| Elongation factor 1 alpha [Hymenolepis microstoma] | + | - | gi\|674593231 | 79 | - | 101285 | 3 | - | 3 | - | 3 | - |
| Elongation factor 1-a, partial [Hymenolepis diminuta] | + | + | gi\|6467323 | 404 | 221 | 29743 | 7 | 5 | 7 | 5 | 5 | 4 |
| Endophilin B1 [Hymenolepis microstoma] | + | + | gi\|674593521 | 113 | 83 | 22457 | 4 | 3 | 4 | 3 | 3 | 2 |
| Enolase [Hymenolepis microstoma] | + | + | gi\|674591125 | 1456 | 164 | 48506 | 30 | 4 | 30 | 4 | 18 | 3 |
| Fatty acid binding protein a [Hymenolepis microstoma] | + | - | gi\|674593527 | 319 | - | 15312 | 5 | - | 5 | - | 4 | - |
| Filamin [Hymenolepis microstoma] | + | + | gi\|674587174 | 482 | 303 | 274339 | 7 | 6 | 7 | 6 | 6 | 6 |
| Four and a half LIM domains protein 2 [Hymenolepis microstoma] | + | - | gi\|674595939 | 393 | - | 65205 | 7 | - | 7 | - | 6 | - |
| Fructose 16 bisphosphate aldolase [Hymenolepis microstoma] | + | - | gi\|674594832 | 895 | - | 39864 | 18 | - | 18 | - | 14 | - |
| Glutamate dehydrogenase [Hymenolepis microstoma] | + | + | gi\|674591570 | 127 | 182 | 58326 | 3 | 4 | 3 | 4 | 3 | 4 |
| Glyceraldehyde-3-phosphate dehydrogenase [Taenia solium] | + | - | gi\|149364041 | 483 | - | 36467 | 10 | - | 10 | - | 7 | - |
| Heat shock 70kDa protein, partial [Mesocestoides corti] | - | + | gi\|1661112 | - | 426 | 70870 | - | 9 | - | 9 | - | 7 |
| Heat shock cognate protein [Echinococcus granulosus] | + | - | gi\|576692679 | 461 | - | 71269 | 8 | - | 8 | - | 7 | - |
| Heat shock protein 60 [Echinococcus multilocularis] | + | + | gi\|674580112 | 317 | 418 | 60896 | 4 | 7 | 4 | 7 | 3 | 6 |
| Heat shock protein 70 [Hymenolepis microstoma] | + | + | gi\|674591003 | 471 | 187 | 66008 | 6 | 4 | 6 | 4 | 4 | 3 |
| Heat shock protein 71 kDa protein [Hymenolepis microstoma] | + | - | gi\|961498834 | 188 | - | 37663 | 4 | - | 4 | - | 4 | - |
| Histone [Echinococcus granulosus] | - | + | gi\|674569431 | - | 113 | 34786 | - | 4 | - | 4 | - | 4 |
| Histone cluster 2 H3c2 [Hymenolepis microstoma] | + | - | gi\|674592388 | 166 | - | 15412 | 4 | - | 4 | - | 4 | - |
| Histone H2A [Echinococcus granulosus] | + | - | gi\|674560634 | 189 | - | 12451 | 4 | - | 4 | - | 3 | - |
| Histone H2B [Echinococcus granulosus] | + | + | gi\|576690363 | 391 | 718 | 13513 | 7 | 11 | 7 | 11 | 6 | 5 |
| Hydrophobic ligand binding protein [Hymenolepis diminuta] | + | - | gi\|9963978 | 451 | - | 8678 | 10 | - | 10 | - | 6 | - |
| Hypothetical transcript [Hymenolepis microstoma] | + | + | gi\|961496618 | 389 | 880 | 94000 | 7 | 16 | 7 | 16 | 6 | 13 |
| Major egg antigen [Hymenolepis microstoma] | - | + | gi\|674587238 | - | 181 | 52787 | - | 4 | - | 4 | - | 4 |
| Myosin heavy chain [Hymenolepis microstoma] | + | + | gi\|674587834 | 1976 | 1048 | 224148 | 35 | 27 | 35 | 27 | 29 | 25 |
| Myosin heavy chain non muscle [Hymenolepis microstoma] | - | + | gi\|961497681 | - | 196 | 232075 | - | 6 | - | 6 | - | 6 |
| Myosin heavy chain, striated muscle [Echinococcus granulosus] | + | + | gi\|576698220 | 1674 | 660 | 227242 | 31 | 20 | 31 | 20 | 23 | 18 |
| Myosin regulatory light chain [Taenia asiatica] | + | - | gi\|124783553 | 138 | - | 19539 | 3 | - | 3 | - | 3 | - |
| NADP dependent malic enzyme [Hymenolepis microstoma] | + | - | gi\|961496084 | 287 | - | 15076 | 5 | - | 5 | - | 3 | - |
| Ndr [Hymenolepis microstoma] | + | - | gi\|674591004 | 193 | - | 40928 | 2 | - | 2 | - | 2 | - |
| Neuronal nitric oxide synthase protein inhibitor [Taenia solium] | + | - | gi\|94556988 | 181 | - | 11501 | 4 | - | 4 | - | 2 | - |
| Nucleoside diphosphate kinase A [Hymenolepis microstoma] | + | - | gi\|674595393 | 136 | - | 17276 | 4 | - | 4 | - | 3 | - |
| Oncosphere protein Tso22a [Taenia solium] | + | - | gi\|59709842 | 599 | - | 42856 | 15 | - | 15 | - | 10 | - |
| Oncosphere protein Tso22e,oncosphere protein Tso22d,oncosphere protein Tso22c,oncosphere protein Tso22b,oncosphere protein Tso22a [Hymenolepis microstoma] | + | - | gi\|674593128 | 306 | - | 38823 | 6 | - | 6 | - | 5 | - |
| Paramyosin [Hymenolepis microstoma] | + | + | gi\|961497969 | 514 | 368 | 100704 | 12 | 8 | 12 | 8 | 9 | 7 |
| PDZ and LIM domain protein Zasp [Hymenolepis microstoma] | + | - | gi\|674593172 | 311 | - | 22078 | 7 | - | 7 | - | 6 | - |
| Phosphoenolpyruvate carboxykinase [Hymenolepis microstoma] | + | - | gi\|674593372 | 451 | - | 70871 | 13 | - | 13 | - | 12 | - |
| Phosphoglycerate kinase 1 [Hymenolepis microstoma] | + | - | gi\|674588583 | 254 | - | 44474 | 6 | - | 6 | - | 5 | - |
| Phosphoglycerate mutase [Hymenolepis microstoma] | + | - | gi\|674586901 | 181 | - | 28784 | 4 | - | 4 | - | 4 | - |
| Procollagen lysine2 oxoglutarate 5 dioxygenase [Hymenolepis microstoma] | - | + | gi\|674595722 | - | 158 | 82716 | - | 6 | - | 6 | - | 5 |
| Protein disulfide isomerase A3 [Hymenolepis microstoma] | + | + | gi\|674589658 | 125 | 139 | 54129 | 3 | 3 | 3 | 3 | 1 | 2 |
| Protein disulfide-isomerase [Echinococcus granulosus] | + | + | gi\|576692553 | 98 | 197 | 55363 | 2 | 5 | 2 | 5 | 2 | 3 |
| Putative 14-3-3 protein [Echinococcus granulosus] | - | + | gi\|62178030 | - | 216 | 28158 | - | 4 | - | 4 | - | 3 |
| Pyruvate dehydrogenase E1 component subunit alpha type I [Echinococcus granulosus] | - | + | gi\|576694194 | - | 83 | 42795 | - | 3 | - | 3 | - | 3 |
| Pyruvate kinase [Echinococcus granulosus] | + | - | gi\|674568864 | 221 | - | 62707 | 4 | - | 4 | - | 4 | - |
| Pyruvate kinase isozymes M1:M2 [Hymenolepis microstoma] | + | - | gi\|674595321 | 222 | - | 113844 | 4 | - | 4 | - | 4 | - |
| Ribosomal protein S3 [Hymenolepis microstoma] | - | + | gi\|674594336 | - | 127 | 26519 | - | 3 | - | 3 | - | 2 |
| Sj-Ts4 protein [Echinococcus granulosus] | + | - | gi\|576693238 | 124 | - | 34195 | 3 | - | 3 | - | 2 | - |
| SOD [Spirometra erinaceieuropaei] | - | + | gi\|37624319 | - | 134 | 16102 | - | 2 | - | 2 | - | 2 |
| Sodium/potassium-transporting ATPase subunit alpha | - | + | gi\|74794482 | - | 640 | 112838 | - | 12 | - | 12 | - | 11 |
| Spectrin alpha actinin [Hymenolepis microstoma] | + | + | gi\|674591524 | 218 | 663 | 282700 | 4 | 20 | 4 | 20 | 5 | 19 |
| Spectrin alpha chain [Echinococcus granulosus] | + | - | gi\|576696380 | 184 | - | 282536 | 7 | - | 7 | - | 7 | - |
| Spectrin beta chain [Hymenolepis microstoma] | - | + | gi\|961498176 | - | 366 | 271653 | - | 12 | - | 12 | - | 12 |
| Succinate dehydrogenase [ubiquinone] flavoprotein subunit [Echinococcus granulosus] | + | - | gi\|576699397 | 300 | - | 71661 | 8 | - | 8 | - | 7 | - |
| Succinyl coenzyme A synthetase alpha subunit [Hymenolepis microstoma] | + | - | gi\|674592910 | 184 | - | 34173 | 4 | - | 4 | - | 3 | - |
| Succinyl-CoA ligase [GDP-forming] subunit alpha [Echinococcus granulosus] | + | - | gi\|576693072 | 146 | - | 35308 | 3 | - | 3 | - | 3 | - |
| Titin [Hymenolepis microstoma] | + | - | gi\|961500888 | 333 | - | 867415 | 5 | - | 5 | - | 5 | - |
| Transketolase [Hymenolepis microstoma] | + | + | gi\|674592527 | 323 | 172 | 68312 | 4 | 4 | 4 | 4 | 3 | 4 |
| Triosephosphate isomerase [Hymenolepis microstoma] | + | - | gi\|674591188 | 438 | - | 27806 | 10 | - | 10 | - | 6 | - |
| Tropomyosin [Hymenolepis microstoma] | + | + | gi\|674593160 | 158 | 164 | 32780 | 5 | 3 | 5 | 3 | 4 | 3 |
| Troponin i [Hymenolepis microstoma] | + | - | gi\|674592091 | 445 | - | 29480 | 8 | - | 8 | - | 7 | - |
| Troponin I 4 [Echinococcus granulosus] | + | - | gi\|576695239 | 204 | - | 30827 | 6 | - | 6 | - | 5 | - |
| Tubulin [Spirometra erinaceieuropaei] | - | + | gi\|421975927 | - | 343 | 50694 | - | 7 | - | 7 | - | 5 |
| Tubulin alpha 1C chain [Hymenolepis microstoma] | - | + | gi\|961496777 | - | 497 | 49832 | - | 12 | - | 12 | - | 8 |
| Tubulin beta 2C chain [Hymenolepis microstoma] | + | + | gi\|674595068 | 315 | 1661 | 50272 | 5 | 34 | 5 | 34 | 3 | 21 |
| Tubulin beta-2 chain | - | + | gi\|29337144 | - | 1202 | 50181 | - | 28 | - | 28 | - | 16 |
| Tubulin beta-3 [Taenia asiatica] | - | + | gi\|124783838 | - | 201 | 26555 | - | 7 | - | 7 | - | 5 |
| Type II collagen B [Hymenolepis microstoma] | - | + | gi\|961498737 | - | 222 | 159482 | - | 4 | - | 3 | - | 3 |
| Zinc transporter zip8 [Hymenolepis microstoma] | + | - | gi\|674592183 | 104 | - | 57259 | 3 | - | 3 | - | 3 | - |
